# FGF Signaling Promotes Lysosome Biogenesis in Chondrocytes via the Mannose Phosphate Receptor Pathway

**DOI:** 10.1111/tra.70013

**Published:** 2025-08-01

**Authors:** Laura Cinque, Maria Iavazzo, Gennaro Di Bonito, Elena Polishchuk, Rossella De Cegli, Carmine Settembre

**Affiliations:** ^1^ Telethon Institute of Genetics and Medicine (TIGEM) Pozzuoli Italy; ^2^ Department of Clinical Medicine and Surgery Federico II University Naples Italy

**Keywords:** chondrocytes, FGF signaling, lysosome, mannose phosphate receptors, MPR trafficking pathway, TFEB

## Abstract

The mannose 6‐phosphate (M6P) pathway is critical for lysosome biogenesis, facilitating the trafficking of hydrolases to lysosomes to ensure cellular degradative capacity. Fibroblast Growth Factor (FGF) signaling, a key regulator of skeletogenesis, has been linked to the autophagy‐lysosomal pathway in chondrocytes, but its role in lysosome biogenesis remains poorly characterized. Here, using mass spectrometry, lysosome immune‐purification, and functional assays, we reveal that RCS (Swarm rat chondrosarcoma cells) lacking FGF receptors 3 and 4 exhibit dysregulations of the M6P pathway, resulting in hypersecretion of lysosomal enzymes and impaired lysosomal function. We found that FGF receptors control the expression of M6P receptor genes in response to FGF stimulation and during cell cycle via the activation of the transcription factors TFEB and TFE3. Notably, restoring M6P pathway—either through gene expression or activation of TFEB—significantly rescues lysosomal defects in FGFR3;4‐deficient RCS. These findings uncover a novel mechanism by which FGF signaling regulates lysosomal function, offering insights into the control of chondrocyte catabolism and the understanding of FGF‐related human diseases.

## Graphical abstract

FGF signaling regulates lysosomal homeostasis in RCS chondrocytes by inducing TFEB/TFE3 nuclear translocation and activation. This promotes the expression of lysosomal genes and mannose 6‐phosphate receptors, enhancing lysosome biogenesis and lysosomal hydrolases delivery. Disruption of this pathway leads to lysosomal swelling, functional impairment, and excessive secretion of hydrolases, underscoring the central role of FGF signaling in lysosomal function.

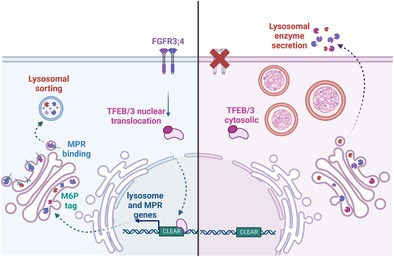

## 1 Introduction

Lysosomes are essential degradative organelles within the cytoplasm, playing critical roles in maintaining cellular homeostasis [1]. Their biogenesis is regulated at the transcriptional level by the microphthalmia/transcription factor E (MiT/TFE) family of transcription factors, which promote lysosomal gene expression in response to extracellular stimuli, such as nutrient starvation [2, 3, 4, 5, 6]. The MiT/TFE family consists of four members: TFEB, TFE3, MITF, and TFEC. Except for the divergent TFEC, all members bind to a palindromic consensus sequence in lysosomal gene promoters, known as the CLEAR (Coordinated Lysosomal Expression and Regulation) site, to enhance lysosome biogenesis and autophagy [4, 6]. TFEB activity is primarily regulated by the mTORC1 kinase, which controls its cytoplasmic‐to‐nuclear shuttling via phosphorylation [1, 2, 3, 4, 5, 6, 7, 8].

At the post‐translational level, lysosome biogenesis is largely governed by the mannose 6‐phosphate (M6P) pathway in the Golgi [9]. Soluble lysosomal enzymes synthesized in the endoplasmic reticulum are transported to the Golgi via the CLN6‐CLN8 [10] complex and subsequently modified with a mannose 6‐phosphate (M6P) residue. This tagging process involves two key enzymes: GNPT (N‐acetylglucosamine 1‐phosphotransferase) in the cis‐Golgi, which adds a GlcNAc‐1‐phosphate group to specific mannose residues on lysosomal enzyme precursors, and NAGPA (N‐acetylglucosamine 1‐phosphodiester α‐N‐acetylglucosaminidase), also known as the uncovering enzyme (UCE), which removes N‐acetylglucosamine (GlcNAc) residues from mannose 6‐phosphate (M6P) precursors in the trans‐Golgi network (TGN), thereby exposing the mannose 6‐phosphate recognition marker necessary for proper lysosomal trafficking [9, 11]. Recently, LYSET (Lysosomal Enzyme Trafficking factor) protein, encoded by the *Tmem251* gene, has been identified as playing a key role in stabilizing and enhancing the activity of GNPTAB [12, 13, 14], thereby supporting the M6P pathway. The M6P‐tagged lysosomal enzymes are recognized by the cation‐dependent mannose‐phosphate receptor (MPR‐CD) or the cation‐independent receptor (MPR‐CI), which deliver the enzymes from the TGN to late endosomes/lysosomes. Upon arrival, the acidic pH in endo‐lysosomes promotes the dissociation of the MPR‐ligand complex, and the recycling of MPRs to the TGN or plasma membrane [9, 11].

Failure in this recognition process results in hypersecretion of lysosomal enzymes into the extracellular space. Loss‐of‐function mutations in genes encoding components of the M6P pathway disrupt lysosome biogenesis, leading to lysosomal storage diseases. An example of such a disorder is Mucolipidosis type II (MLII), a rare inherited condition caused by mutations in the *GNPTAB* gene [9, 11, 15].

Although the basic mechanisms controlling lysosomal function are characterized, their coordination in modulating lysosome activity in response to cellular needs remains poorly understood. This gap is particularly significant in the context of organismal development, where lysosomes may contribute to specific functions.

Fibroblast Growth Factor (FGF) signaling is a known regulator of skeletal development [16, 17]. FGF signaling regulates key functions in chondrocytes, including proliferation, differentiation, and the production of extracellular matrix. Specifically, FGF receptor 3 (FGFR3) is known to inhibit cell cycle progression and promote hypertrophic differentiation. In contrast, FGFR1 and FGFR2 are primarily involved in osteoblast function at the calcified zone of the growth plate. The role of FGFR4 in cartilage remains less well understood [16, 17]. These tyrosine kinase receptors can be activated by 22 distinct ligands, each exerting a variety of functions [17]. We recently demonstrated that the ligand FGF18 promotes lysosome biogenesis and autophagy in chondrocytes through the activation of FGFR3 and FGFR4, at least in part, via TFEB [18, 19, 20]. Deletion of *Fgfr3* and *Fgfr4* in the Swarm Rat Chondrosarcoma (RCS) cell line completely abrogates the effect of FGF ligand on mTORC1‐mediated TFEB regulation, and the analysis of FGFR3;4‐deficient RCS revealed lysosomal swelling and accumulation of undigested substrates, indicating a key role for FGFR3;4 in lysosome homeostasis [19].

In this study, we characterized the lysosomal phenotype in FGFR3;4‐deficient RCS and found that it is primarily a consequence of lysosomal enzyme mis‐targeting, due to defects in the M6P pathway. These findings reveal that FGF signaling regulates the trafficking of lysosomal proteins in RCS chondrocytes, uncovering novel mechanisms by which mutations in FGF signaling may contribute to human diseases associated with FGF mutations.

## 2 Results

### 2.1 Impaired Differentiation and Lysosome Function in RCS Lacking FGF Receptors

**FIGURE 1:**
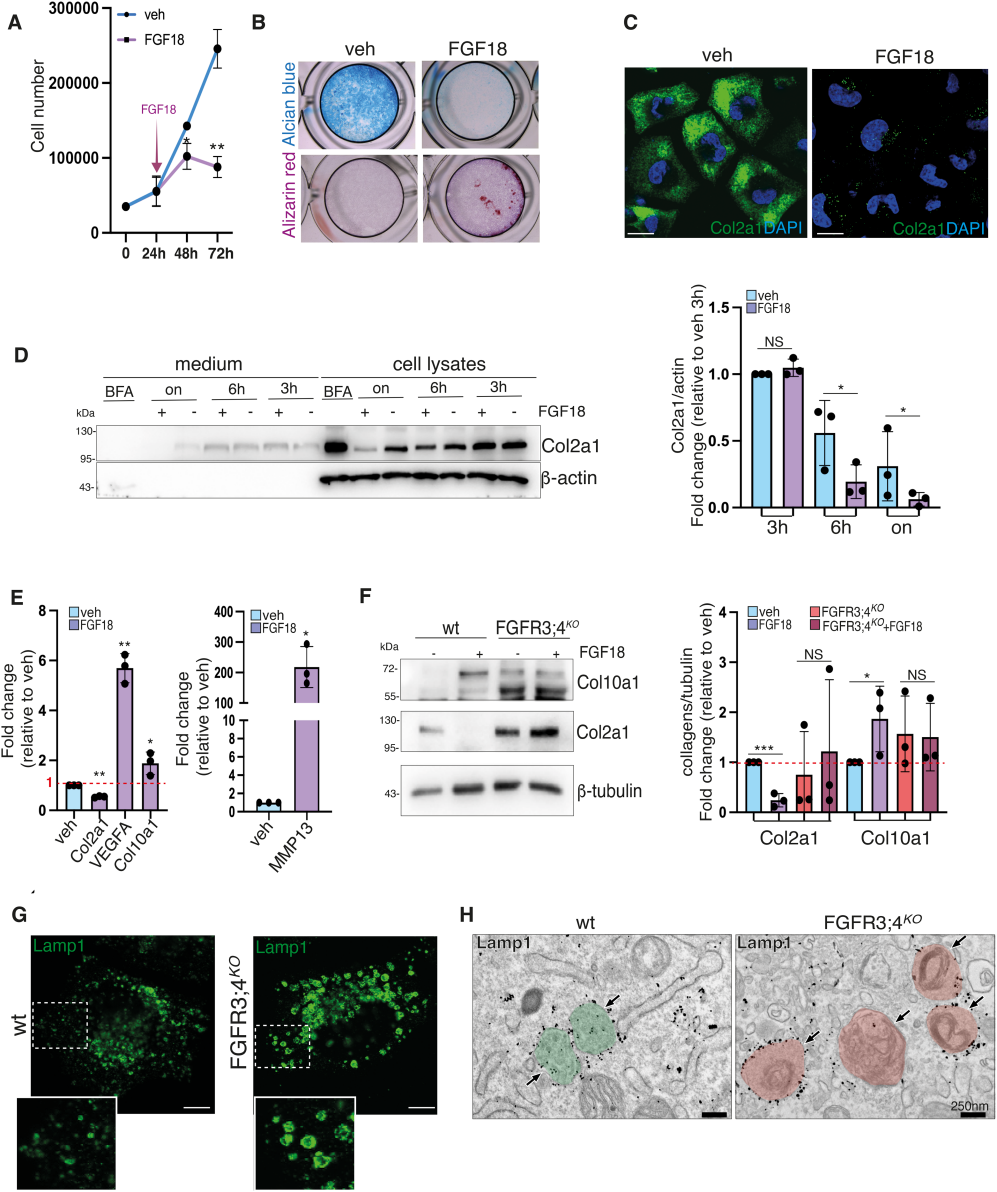
Impaired RCS chondrocyte differentiation and lysosomal dysfunction in cells lacking FGF receptors. (A) Cell number analysis of RCS cell cultures grown for 24 h, and then treated with vehicle (5% ABS) or FGF18 (50 ng/mL) for a further 48 h. *n* = 3 biological replicates ± sem; Unpaired Student's *T*‐test *p < 0.05, **p < 0.005. (B) Alcian blue and Alizarin Red staining of RCS cells treated with vehicle (5% ABS) or FGF18 (50 ng/mL) for 3 days. (C) Col2a1 staining (green) of RCS cells treated with vehicle (5% ABS) or FGF18 (50 ng/mL overnight) showing decreased levels of Col2a1 in FGF18‐treated RCS cells. DAPI (blue) was used to stain nuclei. Scale bar 10 μm (D) Representative Western blot analysis of intracellular and secreted Col2a1 from RCS cells treated with vehicle (5% ABS) or FGF18 (50 ng/mL) for the indicated times (3 h, 6 h, overnight). BrefeldinA (BFA) was used as positive control to inhibit intracellular trafficking. Beta‐Actin was used as a loading control. Graph, quantification of *n* = 3 biological replicates. ± sem; *p < 0.05, Unpaired Student's *T*‐test, NS not significant. (E) qPCR analysis of *Col2a1, VEGFA, Col10a1, and MMP13* in RCS cells treated with vehicle (5% ABS) or FGF18 (50 ng/mL, overnight). Fold change was calculated relative to vehicle. Cyclophillin was used to normalize the data. *N* = 3 biological replicates, ± sem; *p < 0.05, **p < 0.005, Unpaired Student's *T*‐test. (F) Western blot analysis of Col2a1 and Col10a1 in RCS wt and RCS FGFR3;4^KO^ treated with vehicle (5% ABS) or FGF18 (50 ng/mL, overnight). Beta‐tubulin was used as a loading control. Graph, quantification of *n* = 3 biological replicates. ± sem; *p < 0.05, ***p < 0.0005, NS not significant, Unpaired Student's *T*‐test. (G) Immunofluorescence staining of Lamp1 in RCS wt and RCS FGFR3;4^KO^ cells. Insets, magnification of the boxed area showing lysosomal swelling in RCS FGFR3;4^KO^. Scale bar, 5 μm. (H) Immuno‐gold EM analysis of Lamp1 in RCS wt and RCS FGFR3;4^KO^. Arrows indicate lysosomes decorated with Lamp1‐gold nanoparticles. Lysosomes are highlighted in green in RCS wt cells and red in FGFR3;4^KO^ cells. Note the undigested membranes in the FGFR3;4^KO^ lysosomes. Scale bar, 250 nm.

The RCS cell line proliferates and produces glycosaminoglycans and type II collagen (COL2A1), resembling growth plate chondrocytes in the proliferative state. Upon stimulation with Fibroblast Growth Factor 18 (FGF18), RCS cells initiate a differentiation process toward hypertrophic chondrocytes, as evidenced by reduced cell proliferation and glycosaminoglycan deposition (Alcian blue staining), alongside increased matrix calcification (Alizarin Red positive staining) (Figure 1A,B). Furthermore, FGF18 downregulates COL2A1 production (Figure 1C–F) and activates the expression of hypertrophic markers such as collagen type X (*Col10a1*), *Vegf*, and *Mmp13* (Figure 1E,F). Deletion of Fibroblast Growth Factor Receptor 3 (FGFR3) and Fibroblast Growth Factor Receptor 4 (FGFR4) using CRISPR/Cas9 technology (FGFR3;4^KO^ RCS) completely inhibited RCS differentiation response to FGF18 (Figure 1F), indicating an essential role for FGFR3 and FGFR4 in mediating FGF18‐dependent RCS hypertrophic differentiation. FGF18 stimulation induces lysosome biogenesis and autophagy in wild‐type but not in FGFR3;4^KO^ RCS [18, 19] and, notably, FGFR3;4^KO^ RCS exhibited pronounced lysosomal phenotypes even in non‐stimulated conditions. These phenotypes are defined by: (1) enlarged, swollen lysosomes, as highlighted by LAMP1 immunostaining (Figures 1G and S1A,B), and (2) the presence of undigested membranous cellular debris within these swollen lysosomes as shown by electron microscopy coupled to LAMP1 immunogold labeling (Figure 1H). A similar phenotype was observed in different FGFR3;4^KO^ clones (Figure S1A,B) and it can be rescued by reintroducing FGFR3 or FGFR4 proteins (Figure S1C).

Collectively, these observations indicate a critical role for FGF signaling in inducing hypertrophic differentiation and regulating lysosomal function in RCS chondrocytes.

### 2.2 Mis‐Targeting of Lysosomal Proteins in RCS Lacking FGFR3 and FGFR4

**FIGURE 2:**
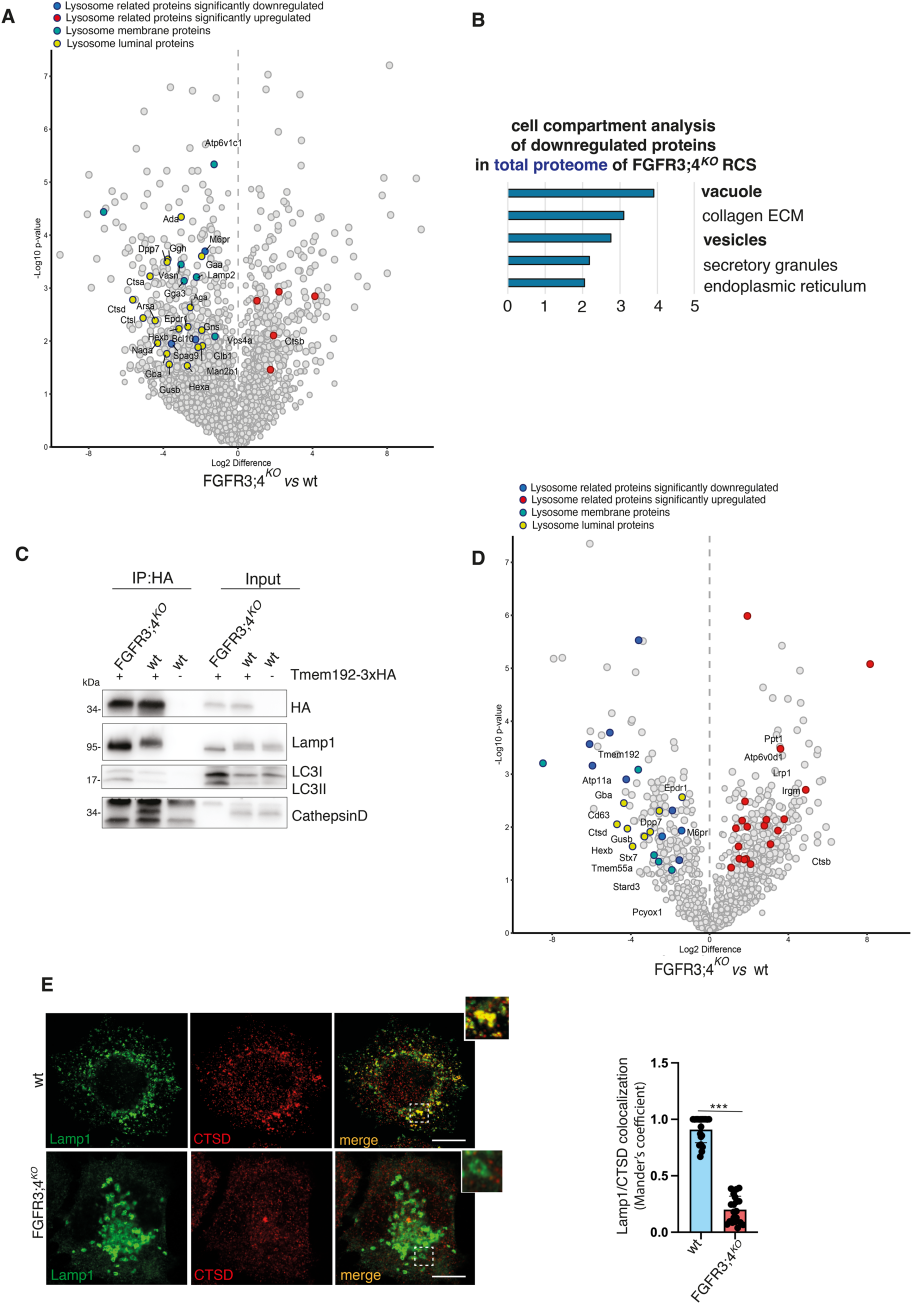
Dysregulated lysosomal profile in RCS chondrocytes lacking FGF receptors. (A) MS proteomic analysis of wt RCS and RCS FGFR3;4^KO^ cells. *n* = 4 biological replicates. (B) Cellular Compartment (CC) enrichment for the downregulated expressed proteins from Figure 2A. (C) Western Blot analysis of TMEM192‐3xHA immune‐isolated lysosomes (Lyso‐IP, see Methods). An anti‐HA antibody was used to IP intact lysosomes, and Lamp1, Cathepsin D, LC3 antibodies were used to analyze the auto‐lysosome protein profile. (D) MS proteomic analysis of TMEM192‐3xHA tagged lysosomes isolated (Lyso‐IP) from wt RCS and RCS FGFR3;4^KO^ cells. *n* = 4 biological replicates. (E) Immunofluorescence staining of Lamp1 and Cathepsin D (CTSD) in wt RCS and RCS FGFR3;4^KO^. Insets show a magnification of the boxed area. Lamp1 in green, Cathepsin D in red. Scale bar, 5 μm. Colocalization analysis of Lamp1 and Cathepsin D in wt RCS and RCS FGFR3;4^KO^, showing decreased colocalization in FGFR3;4^KO^ cells. *n* = 20 cells/genotype. ± sem; ***p < 0.0005, Unpaired Student's *T*‐test.

To investigate the molecular mechanisms underlying this lysosomal dysfunction, we performed mass spectrometry (MS) analysis on whole‐cellular extracts from control and FGFR3;4^KO^ cells. Notably, cellular compartment analysis showed lysosome as the most significantly downregulated category in FGFR3;4^KO^ cells (Tables S1 and S2) (Figure 2A,B), with at least 19 lysosomal luminal proteins significantly downregulated in FGFR3;4^KO^ compared to control RCS. Additional cellular compartments—including the vesicle membrane/endosomes, and organelle subcompartments—were downregulated following the loss of FGFR3 and FGFR4 function in RCS cells (Table S2). Notably, the vesicle membrane/endosome and organelle subcompartment categories include numerous lysosomal and lysosome‐associated pathway proteins, further supporting the conclusion that the lysosomal compartment is among the most significantly affected cellular components.

Next, to obtain a more detailed picture of lysosomal alterations in FGFR3;4^KO^ cells, we employed lysosomal immunoprecipitation (Lyso‐IP) protocol. Immune‐isolated TMEM192‐3xHA‐tagged lysosomes from FGFR3;4^KO^‐TMEM192‐3xHA RCS showed absence of mature Cathepsin D (CTSD) compared to lysosomes isolated from control cells (Figure 2C). MS analysis on lysosomal fractions further demonstrated that multiple lysosomal luminal hydrolases, such as Cathepsin D (CTSD), β‐Glucuronidase (GUSB), Hexosaminidase B (HEXB), and Dipeptidyl‐Peptidase 7 (DPP7), were significantly reduced in FGFR3;4^KO^ lysosomes compared to controls (Figure 2D; Figure S1D; Tables S3 and S4). We also observed alterations in lysosomal membrane proteins, including LAMP1 and ACP2, as well as in lysosome‐associated proteins such as M6PR, IGF2R, and CD63 (Table S4). Moreover, co‐immunostaining with the lysosomal marker LAMP1 confirmed reduced levels of lysosomal CTSD in FGFR3;4^KO^ compared to control cells (Figure 2E).

**FIGURE 3:**
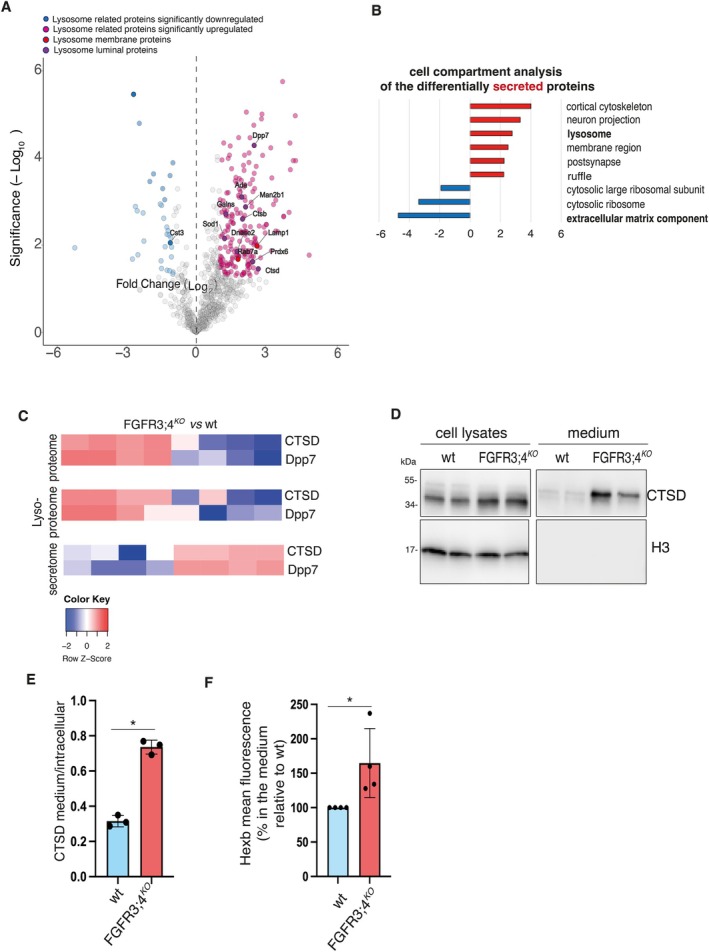
Enhanced secretion of lysosomal hydrolases in FGFR3;4^KO^ cells. (A) MS‐Secretome analysis of the media from wt RCS and RCS FGFR3;4^KO^ (see Methods), showing increased protein secretion in FGFR3;4^KO^ cells. *n* = 4 biological replicates. (B) Cellular Compartment (CC) enrichment for the differentially expressed secreted proteins of MS‐secretome data from Figure 3A. The red and the blue bars are the CC for the incresed and decreased proteins, respectively. (C) Heat maps showing cross analysis of proteins from the total proteome (Figure 2A), lysosome proteome (Figure 2D), and secretome (Figure 3A). (D) Western blot analysis of cell lysates and media fractionated from wt RCS and RCS FGFR3;4^KO^. The immature form of Cathepsin D were analyzed to confirm the secretion of lysosomal hydrolases. H3 was used as loading control. (E) Quantification of *n* = 3 biological replicates for WB experiments showing secreted Cathepsin D normalized to intracellular levels. ± sem; *p < 0.05, Unpaired Student's *T*‐test F. β‐hexosaminidase activity assay in the media of wt RCS and RCS FGFR3;4^KO^. ± sem; *p < 0.05, Unpaired Student's *T*‐test. *n* = 4 biological replicates.

Next, we conducted MS‐based secretome analysis comparing WT and FGFR3;4^KO^ RCS. We observed that FGFR3;4^KO^ cells showed an impaired secretion of important components of the cartilage extracellular matrix, such as collagens and glycoproteins (Figure 3A,B, Tables S5 and S6). Concomitantly, secretome profiling revealed significant enrichment of lysosome proteins among the upregulated categories secreted by FGFR3;4^KO^ RCS (Figure 3A,B, Tables S5 and S6). This observation suggests that protein reduction in the lysosomes and total lysates was at least in part due to an increased secretion into the extracellular space (Figure 3C, Table S7). Accordingly, western blot and enzymatic assays on cellular and medium fractions showed elevated levels of the immature form of CTSD and of HEXB, respectively, in the medium of FGFR3;4^KO^ cells (Figure 3D–F). These results suggest that an elevated secretion of lysosomal proteins contributes, at least in part, to lysosomal dysfunction in FGFR3;4^KO^ RCS chondrocytes.

### 2.3 Reduced Expression of Mannose 6‐Phosphate Receptor Genes in Cells Lacking FGF Signaling

**FIGURE 4:**
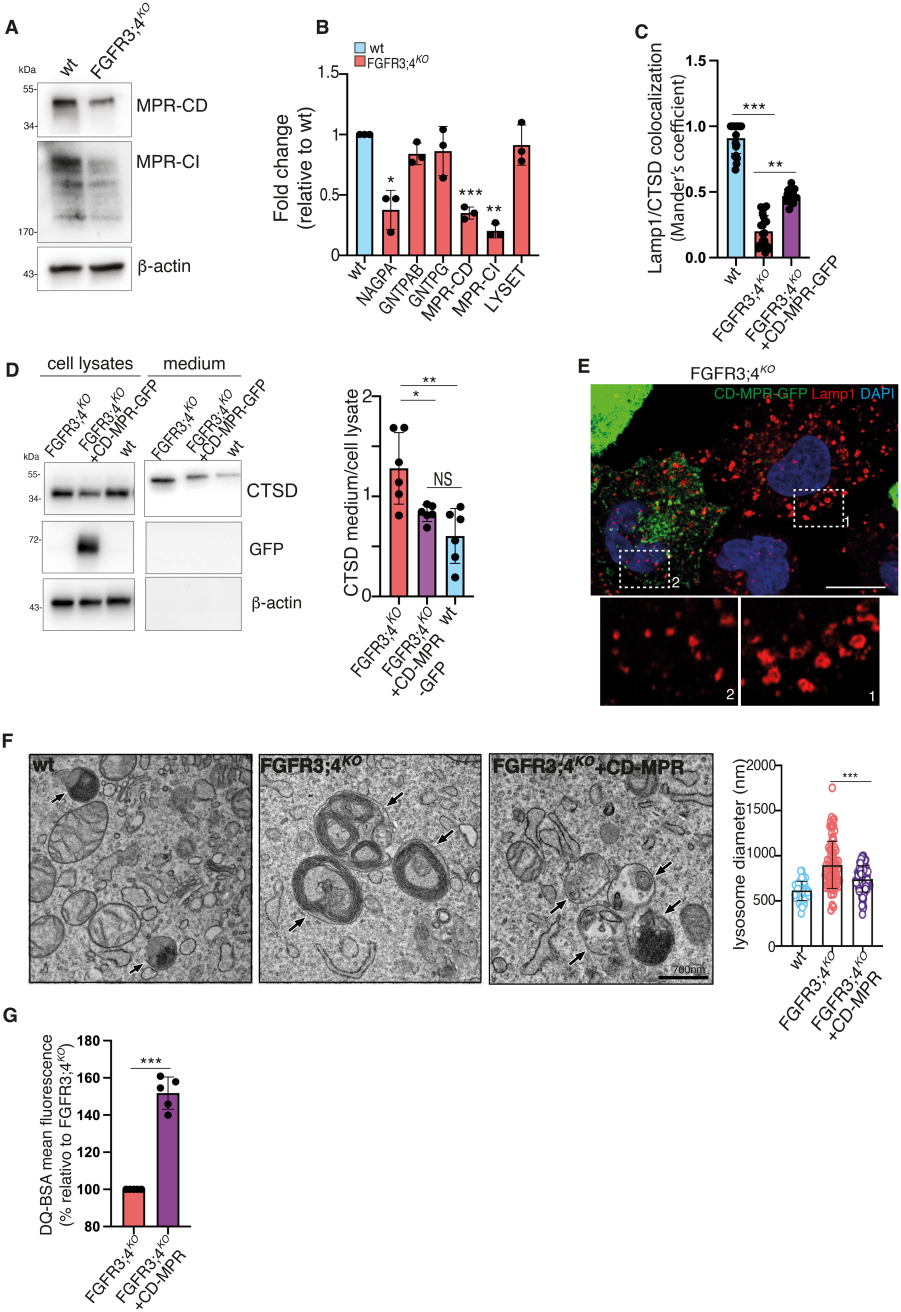
FGFR3 and FGFR4 regulates the mannose 6‐phosphate receptor pathway. (A) Western blot analysis of MPR‐CD and MPR‐CI in wt RCS and RCS FGFR3;4^KO^. Beta‐Actin was used as loading control. (B) qPCR of *Nagpa, Gnptab, Gnptag, Lyset, Mpr‐cd, and Mpr‐ci* gene expression in wt and FGFR3;4^KO^ cells. Values are expressed as fold change relative to wt and normalized to the Cyclophilin gene. ± sem; *p < 0.05; **p < 0.005; ***p < 0.0005, Unpaired Student's *T*‐test. *n* = 3 biological replicates. (C) Colocalization analysis of Lamp1 and Cathepsin D in wt RCS, RCS FGFR3;4^KO^, and RCS FGFR3;4^KO^ overexpressing CD‐MPR‐GFP. *n* = 20 cells/genotype. ± sem; ***p < 0.0005; **p < 0.005, One‐way ANOVA Sidàk's multiple comparison test. (D) Western blot analysis of cell lysates and media fractionated from RCS FGFR3;4^KO^, RCS FGFR3;4^KO^ overexpressing CD‐MPR‐GFP, and wild type RCS. CD‐MPR‐GFP overexpression rescued the hypersecretion of the immature form of Cathepsin D in RCS FGFR3;4^KO^. Beta‐Actin was used as loading control. The Bar graphs represents quantification of secreted CTSD normalized to intracellular proteins level. *N* = 6 biological replicates for CTSD secretion experiments. One‐way ANOVA, Sidak's multiple comparison test *p < 0.05; **p < 0.005; NS not significant. (E) Immunofluorescence analysis of Lamp1 in CD‐MPR‐GFP ‐transfected and un‐transfected RCS FGFR3;4^KO^. CD‐MPR‐GFP in green, Lamp1 in red. The lysosomal phenotype is rescued in CD‐MPR‐GFP overexpressing cells. DAPI (blue) was used to stain nuclei. The lower panels 1 and 2 are magnifications of the boxed areas. Scale bar, 10 μm. (F) TEM analysis of lysosomes in RCS wt, FGFR3;4^KO^ RCS and FGFR3;4^KO^ RCS overexpressing CD‐MPR‐GFP. Arrows indicate lysosomes, which are full of membranes in FGFR3;4^KO^ cells and more electrodense in FGFR3;4^KO^ cells overexpressing CD‐MPR‐GFP, suggesting re‐activated degradation of cargos. Scale bar, 250 nm. Bar graph represents the TEM analysis of lysosomal size in FGFR3;4^KO^ cells overexpressing CD‐MPR‐GFP comparing to FGFR3;4^KO^ RCS. *N* = 25 cells were quantified. ± sem ***p < 0.0005 Unpaired Student's *T*‐test. (G) DQ‐BSA assay in FGFR3;4^KO^ RCS cells and FGFR3;4^KO^ RCS cells overexpressing CD‐MPR‐GFP. ± sem; ***p < 0.0005, Unparied Student's *T*‐Test. *N* = 5 biological replicates.

Lysosomal protein trafficking is regulated by the M6P pathway [9]. FGFR3;4^KO^ RCS exhibited significantly reduced MPR levels, as shown by proteomics and western blot analysis of both the cation‐dependent (MPR‐CD) and cation‐independent (MPR‐CI) forms (Figures 2A,D, 4A). To determine if this reduction resulted from impaired transcription, we performed qPCR analysis to examine the expression levels of genes involved in the MPR pathway, including *Nagpa*, *Gnptab*, *Gnptg*, *Lyset* (encoding TMEM251), *M6pr* (encoding MPR‐CD), and *Igf2r* (encoding MPR‐CI). The analysis revealed a significant downregulation of *Nagpa, M6pr*, and *Igf2r* gene expression in FGFR3;4^KO^ RCS cells compared to wild‐type RCS (Figure 4B).

To evaluate whether MPR deficiency caused the hypersecretion of lysosomal enzymes, we restored MPR‐CD levels by plasmid overexpression. This intervention effectively rescued the lysosomal phenotype of FGFR3;4^KO^ RCS, as demonstrated by multiple lines of evidence. First, we observed an enhanced delivery of Cathepsin D to lysosomes (Figure 4C, Figure S2A). Second, we normalized CTSD secretion levels in the medium (Figure 4D). Third, we observed normalization of lysosomal size (Figure 4E). Fourth, we promoted the clearance of undigested membranous debris, as observed by transmission electron microscopy, indicating the restoration of lysosomal degradative capacity (Figure 4F). Fifth, we obtained increased degradation of the artificial substrate DQ‐BSA (Figure 4G). DQ‐BSA degradation serves as a proxy for lysosomal proteolytic activity, as it depends on the concerted action of lysosomal hydrolases. The observed enhancement in substrate degradation upon MPR restoration indicates that lysosomal enzyme delivery and activity have been successfully re‐established, providing functional validation of lysosomal recovery. Collectively, these findings provide compelling evidence that lysosomal biogenesis defects in FGFR3;4^KO^ RCS cells stem, at least in part, from impaired MPR expression. By rescuing MPR levels, both structural and functional aspects of lysosomes were restored in FGFR3;4‐deficient cells.

### 2.4 Impaired TFEB/TFE3 Nuclear Translocation During the Cell Cycle Accounts for Reduced Levels of MPR Genes in FGFR3;4^KO^ Cells

TFEB and TFE3 transcription factors regulate lysosome biogenesis by binding to CLEAR elements in the promoters of lysosomal genes [3, 4, 5, 6]. ChIP‐seq analysis performed in cells overexpressing TFEB [6, 21, 22, 23, 24, 25] demonstrated that TFEB binds to the promoter of *Nagpa*, *Gnptab*, *M6pr*, and *Igf2r* (Figure S3A,B). FGF18 stimulation enhances TFE3 nuclear translocation in control but not in FGFR3;4^KO^ RCS chondrocytes [19] (Figure S3C) and induced the expression of *M6pr* and *Igf2r* in a TFEB and TFE3 dependent manner, since this effect was lost when FGF18 was applied to TFEB;3^KO^ RCS chondrocytes (Figure S3D).

**FIGURE 5:**
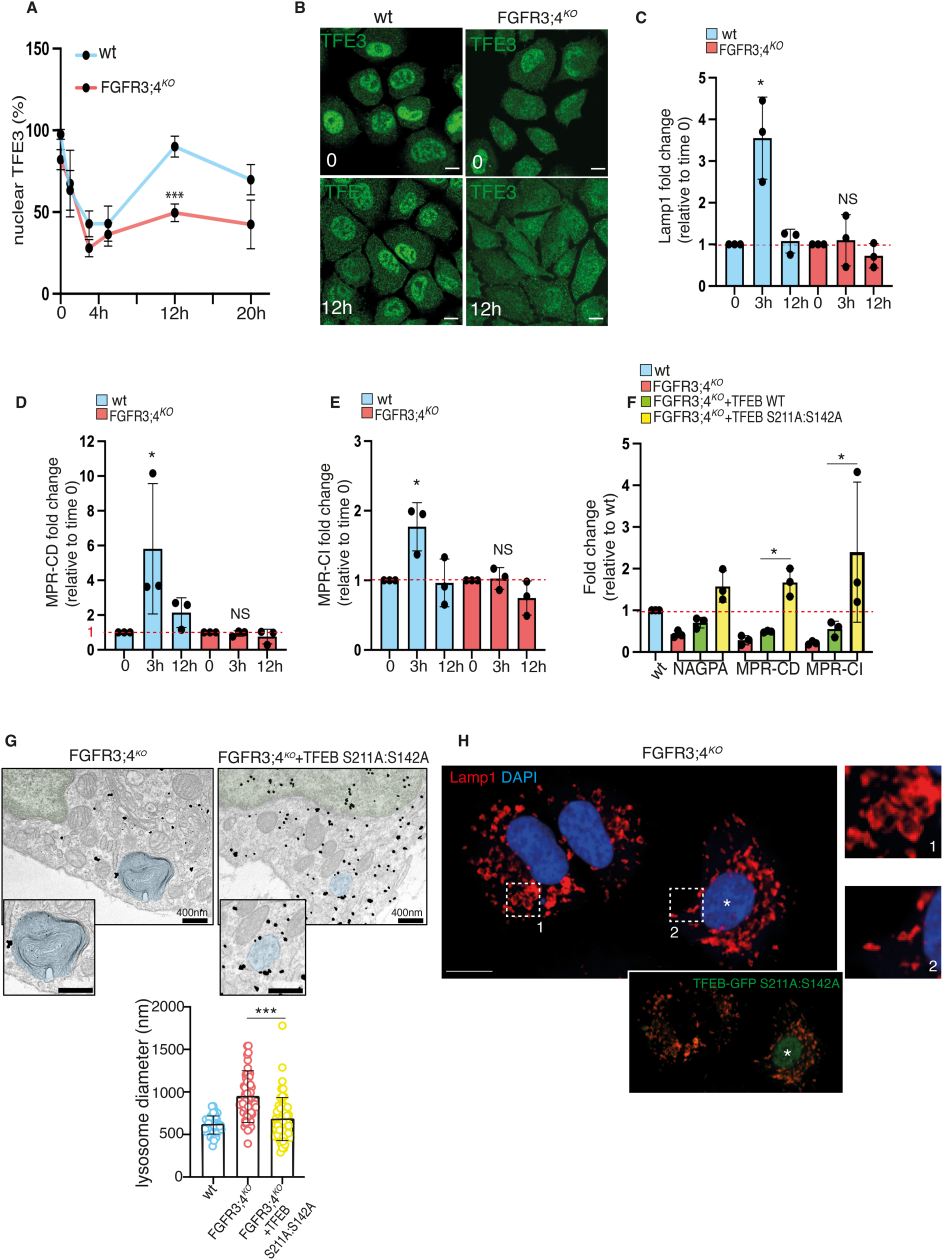
Defective TFEB/TFE3 nuclear translocation during the cell cycle in FGFR3;4^KO^ RCS. (A) Quantification of % of nuclear TFE3 in wt RCS and RCS FGFR3;4^KO^ cells starved for 2 h in HBSS (time 0), and refed with complete medium at different time points (3, 6, 12, and 20 h) until a complete cell cycle replication, showing a slower TFE3 shuttling in FGFR3;4^KO^ RCS. *N* = 3 biological replicates ± sem; ***p < 0.0005, Unpaired Student's *T*‐Test. (B) Representative images of experiment in Figure 5A. Immunofluorescence staining of endogenous TFE3 in wt RCS and RCS FGFR3;4^KO^ cells starved for 2 h in HBSS (time 0), and refed with complete medium for 12 h. Scale bar 10 μm. (C–E) qPCR of *Lamp1*, *Mpr‐cd*, and *Mpr‐ci* genes expression in wt RCS and RCS FGFR3;4^KO^ cells starved for 2 h in HBSS (time 0), and refed with complete medium for different times (3 h, 12 h). Values are fold change relative to time 0 and normalized to the Cyclophilin gene. *n* = 3 biological replicates. ± sem; *p < 0.05, NS not significant, Unpaired Student's *T*‐Test. (F) qPCR of *Nagpa, Mpr‐cd, and Mpr‐ci* gene expression in FGFR3;4^KO^ RCS and in FGFR3;4^KO^ RCS overexpressing either WT TFEB or constitutively active TFEB S211A:S142A, showing the rescue of gene expression upon TFEB overexpression. Values are fold change relative to wt and normalized to the Cyclophilin gene. ± sem; Unpaired Student's *T*‐test *p < 0.05. *n* = 3 biological replicates. (G) Immunogold EM analysis of TFEB (gold nanoparticles) in FGFR3;4^KO^ and FGFR3;4^KO^ RCS overexpressing TFEB S211A:S142A, showing the rescue of the lysosomal phenotype upon TFEB activation. Lysosomes are highlighted in blue, nuclei in green. Scale bar, 400 nm. Bar graph represents the TEM analysis of lysosomal size in wt, FGFR3;4^KO^ RCS and FGFR3;4^KO^ RCS overexpressing TFEB S211A:S142A. RCS wt *N* = 42 cells; RCS FGFR3;4^KO^ *N* = 78 cells; FGFR3;4^KO^ RCS overexpressing TFEB S211A:S142A *N* = 57 cells were quantified. ± sem, ***p < 0.0005 Kruskal test (H) Immunofluorescence analysis of Lamp1 in TFEB S211A:S142A‐overexpressing FGFR3;4^KO^ RCS shows rescue of the lysosomal phenotype upon TFEB overexpression. Panels 1 and 2 on the right show magnification of the boxed areas. Lamp1 in red. The bottom panel shows the same cells stained for TFEB S211A:S142A expression (green). DAPI (blue) was used to stain nuclei. Scale bar 10 μm.

However, under basal conditions, TFEB and TFE3 are cytosolic in both WT and FGFR3;4^KO^ RCS, indicating that they are inactive in unstimulated cells. On the other hand, previous observations indicated that TFEB and TFE3 shuttle from the cytoplasm to the nucleus during the cell cycle [26]. The immunofluorescence analysis of endogenous TFE3 shuttling exhibited clearer signal intensity compared to TFEB in RCS, hence we analyzed TFE3 shuttling during the cell cycle phases in control and FGFR3;4^KO^ RCS cells. FGFR3;4^KO^ RCS cells showed delayed cell cycle progression and impaired TFE3 cytoplasm‐to‐nucleus trafficking compared to WT cells (Figure 5A,B) (Figure S4A–D). The trafficking cycle of TFE3 also aligned with its transcriptional activity, as demonstrated by qPCR analysis of *Lamp1*, a well‐established TFEB/3 target gene. Notably, cyclical fluctuations of *Lamp1* expression were absent in FGFR3;4^KO^ RCS (Figure 5C). The expression levels of *M6pr* and *Igf2r* also changed during the cell cycle in WT but not in FGFR3;4^KO^ RCS chondrocytes (Figure 5D,E). These data indicate that FGF receptors regulate lysosome biogenesis during cell cycle in RCS chondrocytes.

The overexpression of a constitutively active form of TFEB (TFEB‐S211A:S142A) in FGFR3;4^KO^ cells rescued the expression levels of *Nagpa*, *M6pr*, and *Igf2r* (Figure 5F) and, consequently, rescued lysosomal phenotypes, as demonstrated by the analysis of lysosomal size in FGFR3;4^KO^ RCS (Figure 5G,H).

Collectively, these data suggest that FGFR3 and FGFR4 control lysosome biogenesis via TFEB/TFE3‐mediated regulation of mannose 6‐phosphate receptor genes during both cell cycle and in response to FGF stimulation.

## 3 Discussion

Fibroblast growth factor signaling plays a pivotal role in regulating various aspects of chondrocyte biology, including cell proliferation and extracellular matrix homeostasis [16, 17, 18]. In this study, we demonstrate that FGFR3 and FGFR4 regulate lysosome biogenesis and function during the cell cycle by modulating mannose 6‐phosphate receptors expression via TFEB/TFE3 activity in RCS chondrocytes. TFEB and TFE3 are bona fide direct transcriptional regulators of *M6PR* genes. Although this specific aspect was not directly investigated in our study, previous ChIP‐seq experiments have consistently demonstrated TFEB and TFE3 occupancy at CLEAR elements located within the promoters of mannose 6‐phosphate receptor genes [6, 21, 22, 23, 24]. Disruption in FGF‐TFEB/TFE3‐MPR signaling axis lead to increased secretion of lysosomal proteins and impaired lysosomal function. We found that FGF signaling facilitates TFEB nuclear translocation in RCS, and cells deficient in FGFR3 and FGFR4 fail to promote TFEB/TFE3 nuclear translocation in response to FGF stimulation [19] and during the cell cycle progression. Our data suggest that FGF signaling independently modulates both the cell cycle progression and TFEB activity via distinct molecular mechanisms, underscoring its multifaceted role in coordinating cellular proliferation and lysosomal homeostasis. The observed regulation of lysosome protein trafficking during the cell cycle by FGF signaling is nonetheless novel and intriguing. Chondrocytes live embedded into a matrix that is rich in collagens and glycosaminoglycans, whose main intracellular degradation is mediated by lysosomes [27]. However, to our knowledge whether lysosomal enzyme secretion plays physiological roles during chondrogenesis is unknown. An intriguing hypothesis could be that once secreted lysosomal enzymes participate in matrix remodeling during cartilage growth [27].

We observed that FGFR3;4^KO^ cells have alterations in luminal lysosomal enzymes, but also changes in lysosomal proteins that are not typically associated with the mannose 6‐phosphate (M6P)‐dependent pathway, such as membrane proteins. This is consistent with the broad impact of TFEB/TFE3 on lysosome biogenesis, but can also be a secondary consequence of a reduced degradative capacity of the lysosomes. Multiple M6P‐independent pathways contribute to the regulation of trafficking of lysosomal hydrolases, such as the LIMP2‐mediated pathway that controls GBA trafficking [28], the sortilin‐1 trafficking route [29, 30], the LDL receptor/LDL receptor‐related protein 1 (LRP1) pathway [31] and the CLN3‐mediated route [32]. Notably, despite the impairment of GBA trafficking, LIMP2 levels were normal in FGFR3;4^KO^ cells (Figure S2B). Nevertheless, the significant rescue observed in FGFR3;4^KO^ cells upon reintroducing the MPR‐CD protein strongly suggests that the M6PR pathway is a major contributor to the lysosomal phenotype in FGFR3;4^KO^ cells. These data warrant further investigation, with the caveat that cell‐context specificity influences both TFEB regulation of lysosomes and activities of the enzyme trafficking pathways.

Mutations in genes working in the M6P pathway underlie genetic disorders such as Mucolipidosis type II (MLII), which is caused by mutations in *GNPTAB* [9, 11, 15]. MLII is a lysosomal storage disorder characterized by a range of clinical symptoms, including growth retardation and skeletal abnormalities [11]. Recently, another gene, *LYSET* (Lysosome Enzyme Trafficking Factor), was identified. *LYSET* plays a critical role in the trafficking of lysosomal enzymes by regulating GNPTAB protein stability and activity [12, 13, 14]. Notably, mutations in the human *LYSET* gene (*TMEM251*) are associated with skeletal dysplasia and severe short stature [33], highlighting the critical role of a functional M6P pathway in skeletal development. We speculate that the FGF‐mediated control of lysosome biogenesis through the M6P pathway plays a crucial role in activating the catabolic program within the cartilage. The activation of lysosomal degradation pathways might ensure the efficient turnover of cellular components, such as the endoplasmic reticulum [19], enabling the proper cellular remodeling and energy flow needed for the structural and functional changes that are required, for example, during transition to chondrocyte hypertrophy. Hence, the lysosomal dysfunction due to M6P pathway alteration observed in FGFR3;4^KO^ cells may contribute to the skeletal phenotypes associated with alterations in FGF signaling and to the development of diseases, particularly those involving skeletal abnormalities.

## 4 Materials and Methods

### 4.1 Cell Culture, Transfections, Chemicals, and Plasmids

The RCS chondrocyte cell line was cultured in DMEM (Gibco) supplemented with 10% Fetal Bovine Serum (FBS from Euroclone) and 1% pen/strep. In FGF18 experiments, vehicle‐treated cells were cultured in DMEM supplemented with 5% Adult Bovine Serum (ABS from Bio‐Techne) and 1% pen/strep. FGFR3;4^KO^ and TFEB;3^KO^ RCS clones were generated by Crispr/Cas9 technology as previously reported [19].

For transfection experiments, cells were transfected with Lipofectamine LTX and Plus reagent (Invitrogen) for 48 h following a reverse transfection protocol.

Plasmids: TMEM192‐HA was from D. Sabatini's lab (Whitehead Institute, MIT Boston). TFEB‐WT‐GFP, TFEB‐S211A:S142A‐GFP were from A. Ballabio's lab (TIGEM), and M6PR‐GFP was purchased from Origene. hFGFR3 and hFGFR4 plasmids were from Origine, the GFP was cloned by In‐fusion cloning protocol (Takara).

Chemicals: FGF18 ligand (50 ng/mL) was from Peprotech. Brefeldin A (BFA) (Sigma B7651) was used at 10 μg/mL for 3 h to inhibit ER to Golgi trafficking. All experiments including collagen measurements were preceded by ascorbic acid treatment (50 ng/mL for the time of treatment).

### 4.2 Immunofluorescence

RCS chondrocytes were fixed for 15 min in 4% PFA in PBS and permeabilized for 20 min in blocking buffer (0.05% (w/v) saponin, 0.5% (w/v) BSA, 50 mM NH_4_Cl and 0.02% NaN_3_ in PBS). Cells were incubated in a humid chamber for 1 h at room temperature with primary antibodies (Lamp1, Abcam ab24170 1:200; Cathepsin D, Santa Cruz Biotechnology sc‐374 381 1:100; Col2a1, Rockland 600‐401‐104‐0.1 1:50), washed three times in PBS, incubated for 1 h at room temperature with the secondary (Alexa fluor‐labeled 1:400) antibodies, washed again three times in PBS, incubated for 20 min with 1 μg/mL Hoechst 33342, and finally mounted in Mowiol. All confocal images were acquired using a slice thickness of 0.5 μm and the LSM 880 confocal microscope equipped with a 63 × 1.4 numerical aperture oil objective.

*For TFE3 immunofluorescence*: RCS chondrocytes were fixed for 15 min in 4% PFA in PBS and permeabilized for 30 min in 0.02% Triton X‐100 in PBS. Cells were incubated in a humid chamber for 1 h in Blocking Buffer (0.1% Triton X‐100, 10% goat serum in PBS) and then with primary antibodies overnight at 4°C (TFE3, Sigma‐Aldrich HPA023881 1:200) diluted in 0.1% Triton X‐100, 5% goat serum in PBS. Alexa‐fluor conjugated secondary antibodies (1:400) were incubated for 1 h at room temperature in 0.1% Triton X‐100, 1% goat serum in PBS. Nuclei were stained with DAPI 1:1000 in PBS for 20 min at room temperature. Cells were washed with PBS, once in MilliQ water, and mounted with Mowiol. All images were captured using a LSM 880 confocal microscope equipped with a 63 × 1.4 numerical aperture oil objective.

### 4.3 Western Blotting

RCS chondrocytes were washed twice with PBS and then scraped in RIPA lysis buffer supplemented with PhosSTOP and EDTA‐free protease inhibitor tablets, 1× final concentration (Roche, Indianapolis, IN, USA). Cell lysates were incubated on ice for 20 min, then the soluble fraction was isolated by centrifugation at 14000 rpm for 20 min at 4°C. Total protein concentration in cellular extracts was measured using the colorimetric BCA protein assay kit (Pierce Chemical Co, Boston, MA, USA). Protein extracts, separated by SDS‐PAGE and transferred onto PVDF, were probed with primary antibodies overnight against beta‐actin (Novus Biologicals NB600‐501 1:5000), LC3 (Novus Biologicals NB100‐2220 1:1000), Lamp1 (Abcam 24 170 1:1000), beta‐tubulin (Sigma T8660 1:10000) Histone3 (EMD Millipore 07‐690 1:5000), Cathepsin D (Santa Cruz Biotechnology sc‐374 381 1:1000), GFP (Novus NB600‐308 1:1000), FGFR3 (Santa Cruz Biotechnology sc‐123 1:500); FGFR4 (Santa Cruz Biotechnology sc‐124 1:500), HA (Sigma H6908 1:1000), Filamin A (Cell Signaling Technology 4762S 1:5000), MPR‐CD (Abcam ab134153 1:1000), and MPR‐CI (Abcam ab124767 1:1000). Proteins of interest were detected with HRP‐conjugated goat anti‐mouse or anti‐rabbit IgG antibody (1: 2000, Vector Laboratories) and visualized with the ECL Star Enhanced Chemiluminescent Substrate (Euroclone) according to the manufacturer's protocol. The Western blotting images were acquired using the Chemidoc‐lt imaging system (UVP).

*For Col2a1 and Col10a1 Western blotting*: 200 × 10^3^ RCS cells were plated in 12‐well plates and treated the next day with FGF18 (50 ng/mL) and ascorbic acid (50 ng/mL). Cells were lysed in Triton X lysis buffer (NaCl 150 mM, EDTA 1 mM, Tris HCl pH 7.5 25 mM, Triton X‐100 1%) supplemented with PhosSTOP and EDTA‐free protease inhibitor tablets 1X final concentration (Roche, Indianapolis, IN, USA), gently rocked on ice for 10 min, scraped, and frozen/thawed using liquid nitrogen. Lysates were centrifuged for 15 min at 13200 rpm at 4°C. Supernatants were quantified using the colorimetric BCA protein assay kit (Pierce Chemical Co, Boston, MA, USA). Protein extracts, separated by SDS‐PAGE and transferred onto nitrocellulose membranes, were probed with primary antibodies in 5% milk overnight against Col2a1 (Hybridoma Bank II6B3 1:1000) or Col10a1 (Abcam ab58632 1:1000). Proteins of interest were detected with HRP‐conjugated goat anti‐mouse or anti‐rabbit IgG antibody (1:2000, Vector Laboratories) and visualized with the ECL Star Enhanced Chemiluminescent Substrate (Euroclone) according to the manufacturer's protocol. The Western blotting images were acquired using the Chemidoc‐lt imaging system (UVP).

### 4.4 Lyso‐IP

A total of 10 × 10^5^ TMEM‐192‐3xHA RCS cells were plated in a p150 cm dish, harvested after trypsinization, and cell pellets were resuspended in Lyso IP Buffer (25 mM KCl, 50 mM KH_2_PO_4_, 50 mM K_2_HPO_4_ pH 7,2) supplemented with PhosSTOP and EDTA‐free protease inhibitor tablets, 1X final concentration (Roche, Indianapolis, IN, USA). Cells were homogenized with homogenizer chamber and then centrifuged 10 min at 1500 g at 4°C to remove nuclei and unbroken cells. Supernatants were quantified using the colorimetric BCA protein assay kit (Pierce Chemical Co, Boston, MA, USA) and 1% was used as Input fraction. Supernatants were incubated with anti‐HA magnetic beads in Lyso IP buffer overnight at 4°C on a rotator. IP fractions were washed 3 times with Lyso IP buffer for 10 min at 4°C on the rotator, and 5 times with Lyso IP washing buffer (25 mM KCl, 50 mM KH_2_PO_4_, 50 mM K_2_HPO_4_, 300 mM NaCl pH 7,2) for 5 min at room temperature on the rotator. Samples were resuspended in 1x SB, denatured for 10 min at 95°C, and analyzed by SDS‐PAGE or processed for MS‐proteomics. Data analysis was done with MaxQuant 1.6.1 with standard parameters and activated LFQ quantification. Differentially abundant proteins were detected by 5% FDR‐corrected *t*‐tests performed with Perseus 1.6.2.2. The mass spectrometry proteomics data have been deposited to the ProteomeXchange Consortium via the PRIDE [34] partner repository with the dataset identifier PXD063029.

### 4.5 MS‐Proteomics

Proteome preparation was done using the iST sample preparation kit 96x from Preomics. Peptides were separated on a reverse phase column (50 cm, packed in‐house with 1.9‐μm C18‐ Reprosil‐AQ Pur reversed‐phase beads) (Dr Maisch GmbH) over 120 min single‐run gradients at a flowrate of 350 nL on an EASY‐nLC 1200 system (Thermo Fisher Scientific) and analyzed by electrospray tandem mass spectrometry on a QExactive HFX (Thermo Fischer Scientific) using HCD based fragmentation. MS data was acquired using a data dependent top‐15 method with maximum injection time of 20 ms, a scan range of 300–1650th, and an AGC target of 3e6. Survey scans were acquired at a resolution of 60 000. Resolution for HCD spectra was set to 15 000 with maximum ion injection time of 28 ms and an underfill ratio of 30%. Dynamic exclusion was set to 20s. Sequencing was performed via higher energy collisional dissociation fragmentation with a target value of 1e5, and a window of 1.6th. Raw mass spectrometry data were processed with MaxQuant version 1.5.5.2 using default settings (FDR 0.01, oxidized methionine (M) and acetylation (protein N‐term) as variable modifications, and carbamidomethyl I as fixed modification). Label free quantitation (LFQ) and “Match between runs” were enabled. Bioinformatics analysis was performed with Perseus 1.5.4.2. Annotations were extracted from UniProtKB, Gene Ontology (GO), and the Kyoto Encyclopedia of Genes and Genomes (KEGG). The mass spectrometry proteomics data have been deposited to the ProteomeXchange Consortium via the PRIDE [34] partner repository with the dataset identifier PXD063031.

### 4.6 DQ BSA Assay

DQ Green BSA (D12050 Thermo Fisher) was incubated at 1 μg/mL in the dark for 15 min at 37°C. Cells were washed three times with PBS 1×, collected and fluorescence was analyzed by FACS Accuri C6, 10 000 events were collected.

### 4.7 Beta‐Hexosaminidase Assay in the Medium

A total of 50 × 10^3^ RCS cells were seeded in a 96‐well plate in a volume of 100 μL of full medium. The day after, a beta‐hex activity plate was prepared dispensing 17.86 μL of 6 mM beta‐hex substrate into a black 96‐well plate. Media from 96‐well cell plate was transferred to the beta‐hex activity plate containing the activity assay substrate. The activity assay plate was incubated in a Jitter bug at 37°C for 15 min with 1000 rpm shaking. Using a multichannel pipettor, 35.71 μL of beta‐hex stop solution was added to the activity assay plate. The plate was read on SpectraMax i3 at 365 nm excitation and 440 emission.

### 4.8 Proteins Precipitation From Medium

For secretome experiments, 150 × 10^3^ RCS cells were plated in a 12‐well plate. Cells were incubated for 20 min with Optimem (Gibco), washed with PBS1x, and incubated with 500 μL of Optimem (Gibco) for 24 h. Media were harvested and protein precipitation was performed with Amicon Ultra 3 K columns (Millipore). Media were centrifuged for 5 min at 4°C to remove cell debris; then supernatants were centrifuged using Amicon columns for 40 min at 14000 g at 4°C. Concentrated proteins were recovered with reverse spin for 5 min at 1000 g at 4°C. Samples were resuspended with 2x SB, denatured, and separated by SDS‐PAGE.

Samples for MS‐proteomics analysis were precipitated overnight with 4 volumes of acetone, and the day after processed for the MS experiment. Data analysis was done with MaxQuant 1.6.1 with standard parameters and activated LFQ quantification. Differentially abundant proteins were detected by 5% FDR‐corrected *t*‐tests performed with Perseus 1.6.2.2. The mass spectrometry secretome data have been deposited to the ProteomeXchange Consortium via the PRIDE [34] partner repository with the dataset identifier PXD063036.

### 4.9 Alcian Blue/Alizarin Red Staining

Staining of sulfated glycosaminoglycan was performed using Alcian blue dye. 5 × 10^3^ cells were plated in 96‐well plates, washed once with PBS, and fixed for 2 min with ice cold methanol at −20°C. RCS chondrocytes were incubated overnight with 0.1% Alcian Blue in 0.1 N HCl.

Measurement of mineralization was performed by Alizarin Red dye. 5 × 10^3^ RCS chondrocytes were plated in 96‐well plate, washed once with PBS1X and then fixed with PFA 4%, 10 min. Cells were incubated for 1 h with 0.2% Alizarin Red in H_2_O, pH 6.4.

Cells were washed twice with MQ water, dried, and analyzed using Leica M205FA Stereomicroscopy.

### 4.10 Transmission electron Microscopy

For EM analysis, cells and growth plates were fixed in 1% glutaraldehyde in 0.2 M HEPES buffer and then post‐fixed in uranyl acetate and in OsO_4_. After dehydration through a graded series of ethanol, tissue samples were cleared in propylene oxide, embedded in Epoxy resin (Epon 812), and polymerized at 60°C for 72 h. From each sample, thin sections were cut with a Leica EM UC6 ultramicrotome and images were acquired using a FEI Tecnai‐12 (FEI, Einhoven, The Netherlands) electron microscope equipped with a Veletta CCD camera for digital image acquisition.

*For immunogold‐EM staining of Lamp1 and TFEB S211A:S142A‐GFP*: Fixation and immunogold detection of fluorescent protein for electron microscopy was performed using an anti‐Lamp1(Abcam 24 170) and an anti‐GFP (Abcam 13 970) antibodies. RCS chondrocytes expressing Lamp1 or TFEB S211A:S142A‐GFP were labeled using the protocol previously described (Polishchuk et al., 2003). After immunolabeling, cells were embedded in Epon‐812 and cut using a Leica EM FC7 ultramicrotome. EM images were acquired using a FEI Tecnai‐12 electron microscope. Morphometric analyses were performed using iTEM software (Olympus SIS).

### 4.11 Cell Cycle Analysis

A total of 1 × 10^6^ RCS wt and RCS FGFR3;4^KO^ were synchronized with 2 h of HBSS (time 0) and refed at different time points (3, 6, 12, and, 24 h). Cells were collected in 500 μL of PBS1x and fixed in 4,5 mL of cold EtOH 70% at −20°C overnight. The next day, cell pellets were washed with PBS 1x, DNA was extract with DNA extraction buffer (0.2 M Na_2_HPO_4_, 0.1% Triton X‐100 v/v. pH 7.8) and stained with propidium iodide (PI, 200 μg/mL in PBS, 2 mg of DNase free RNase; P4170 Sigma Aldrich) for 30 min at room temperature in the dark. All samples were analyzed on a FACSAria III machine (BD Biosciences). A total of 30.000 events/genotype/treatment were analyzed.

### 4.12 qRT‐PCR

Cultured RCS chondrocytes were harvested for RNA extraction using RNeasy Mini Kit (Cat No./ID: 74106 (250), Qiagen) according to the manufacturer's protocol. 1 μg of total RNA was used for reverse transcription using QuantiTect Reverse Transcription Kit (Qiagen) according to the manufacturer's instructions. qRT‐PCR was performed in triplicate using Light Cycler 480 SYBER Green I Master (Roche) and analyzed by Light Cycler 480 (Roche). The Ct values were normalized to Cyclophillin and the expression of each gene was represented as deltaCt relative to control. All primers used correspond to rat sequences:

NAGPA.

FW: GCTTATACTCTTCCACGCGG.

REV: CTGAGGGGTAACTGGCTAGG.

GNTPAB.

FW: CCTATACCTGCCTATCCCACA.

REV: CCCAGCAATGTTGTCTCTGT.

GNTPG.

FW: GTGTCTGAACCAAGCACCTG.

REV: GCAACTTCTCATAGCCCTGC.

MPR‐CD.

FW: CCTGCAATCGACACACACTC.

REV: ATGACAAGGAGGATGGAGCC.

MPR‐CI.

FW: AGTCAGAAAACTGCCGGTGA.

REV: CGAAGTAATGCACACAGTCTGT.

Col2a1.

FW: CCGATCCCCTGCAGTACATG.

REV: TGCTCTCGATCTGGTTGTTC.

Col10a1.

FW: AGCTCACGGAAAATGACCAG.

REV: GTTCTAAGCGGGGGATTAGG.

VEGFA.

FW: GTGGAAGAAGAGGCCTGGTA.

REV: CACACACACAGCCAAGTCTC.

MMP13.

FW: TGACTTGTGTGACAGGAGCT.

REV: TAAGGAAAGCAGGGAAGGGG.

LAMP1.

FW: AACCCCAGTGTGTCCAAGTA.

REV: GTCTGACAAAGATGTGCTCCT.

LYSET.

FW: ATGGGATGGATTGGAGTGGG.

REV: CCGAGCTTTCAAGGAGTGTG.

## 5 Statistics

Results were presented as bar graphs indicating standard deviation (SD) (*n* ≥ 3). Statistical analysis was performed using an unpaired, two‐tailed Student's *t*‐test, or One‐way anova, post hoc test with sidak multiple comparison test where required. *p*‐values < 0.05 were considered statistically significant.

## Author Contributions

Laura Cinque performed most of the experiments. Maria Iavazzo and Gennaro Di Bonito performed cell assays and immunofluorescence staining. Elena Polishchuk performed electron microscopy analysis. Rossella De Cegli performed MS‐secretome data analysis. Carmine Settembre and Laura Cinque wrote the paper and prepared the figures.

## Ethics Statement

The authors have nothing to report.

## Conflicts of Interest

The authors declare no conflicts of interest.

## Peer Review

The peer review history for this article is available at https://www.webofscience.com/api/gateway/wos/peer‐review/10.1111/tra.70013.

## Supporting information


**Figure S1.** (A) Western blot analysis of FGFR3 and FGFR4 protein levels in RCS wild type (wt) and FGFR3;4^KO^ RCS clones. Beta‐actin was used as loading control. (B) Immunofluorescence analysis of Lamp1 (red) and Lamp2 (green) in RCS wild type (wt) and FGFR3;4^KO^ RCS clones. DAPI (blue) was used to stain nuclei. Scale bar 10 μm. (C) Immunofluorescence analysis of Lamp1 (red) in RCS FGFR3;4^KO^ transfected with pEGFP, FGFR3‐GFP and FGFR4‐GFP plasmids (green). Insets showed higher magnification of lysosomes. DAPI (blue) was used to stain nuclei. Scale bar 10 μm. (D) Cellular Compartment (CC) enrichment for the downregulated expressed proteins from Figure 2D.
**Figure S2.** (A) Immunofluorescence staining of Lamp1 (green) and Cathepsin D (CTSD, red) in wt RCS, RCS FGFR3;4^KO^, and RCS FGFR3;4^KO^ overexpressing CD‐MPR‐GFP (magenta). Magnification of the boxed areas showing lysosome and Cathepsin D colocalization are shown on the right. Scale bar 10 μm. (B) Western blot analysis of LIMP2 protein in RCS wt and RCS FGFR3;4^KO^ showing no significant differences. *N* = 4 biological replicates. FilaminA was used as loading control. Student unpaired *T*‐test NS not significant.
**Figure S3.** (A, B) IGV snapshots showing TFEB binding sites (indicated in dark blue) on the *M6PR* and *IGF2R* promoters, as identified by ChIP‐seq analysis [6]. Graphical representation of TFEB‐binding site sequence in the promoter of *M6pr* and *Igf2r* gene from Chip‐seq experiment [25]. (C) Immunofluorescence staining of TFE3 in RCS wt treated with vehicle (5% ABS) and FGF18 (50 ng/mL overnight). Bar graph represents the quantification of % of cells with nuclear TFE3. ± sem; Unpaired Student’s *T*‐test ***p < 0.0005. *n* = 3 biological replicates. (D) qPCR of *Mpr‐cd* and *Mpr‐ci* genes in RCS wild type (wt) and TFEB;3^KO^ RCS treated with vehicle (5% ABS) or FGF18 (50 ng/mL, overnight). *N* = 3 biological replicates ± sem. One‐way ANOVA, Sidàk’s multiple comparison test *p < 0.05; **p < 0.005; NS not significant.
**Figure S4.** (A‐C) Quantitative analysis of cell cycle distribution (%) obtained by FACS analysis of cell cycle with propidium iodide (PI, 200 μg/mL) in RCS wt and RCS FGFR3;4^KO^ at different time points (0, 3, 6, 12, and 24 h). 30.000 events/genotype/time point were count. (D) Representative overlay of cell cycle histograms analyzed by PI in RCS wt and RCS FGFR3;4^KO^ at 12 h of refeeding.

Table S1.

Table S2.

Table S3.

Table S4.

Table S5.

Table S6.

Table S7.

## Data Availability

The proteomic data that support the findings of this study have been deposited in PRIDE under accession codes PXD063029 (http://www.ebi.ac.uk/pride/archive/projects/PXD063029), PXD063031 (http://www.ebi.ac.uk/pride/archive/projects/PXD063031), and PXD063036 (http://www.ebi.ac.uk/pride/archive/projects/ PXD063036). All other data supporting the findings of this study are available from the corresponding authors on reasonable request.
