# Supplementary material for: FGF Signaling Promotes Lysosome Biogenesis in Chondrocytes via the Mannose Phosphate Receptor Pathway
Source: Traffic. 2025 Aug 1;26(7-9):e70013. doi: 10.1111/tra.70013 (PMC12314853; doi:10.1111/tra.70013)

Figure 1

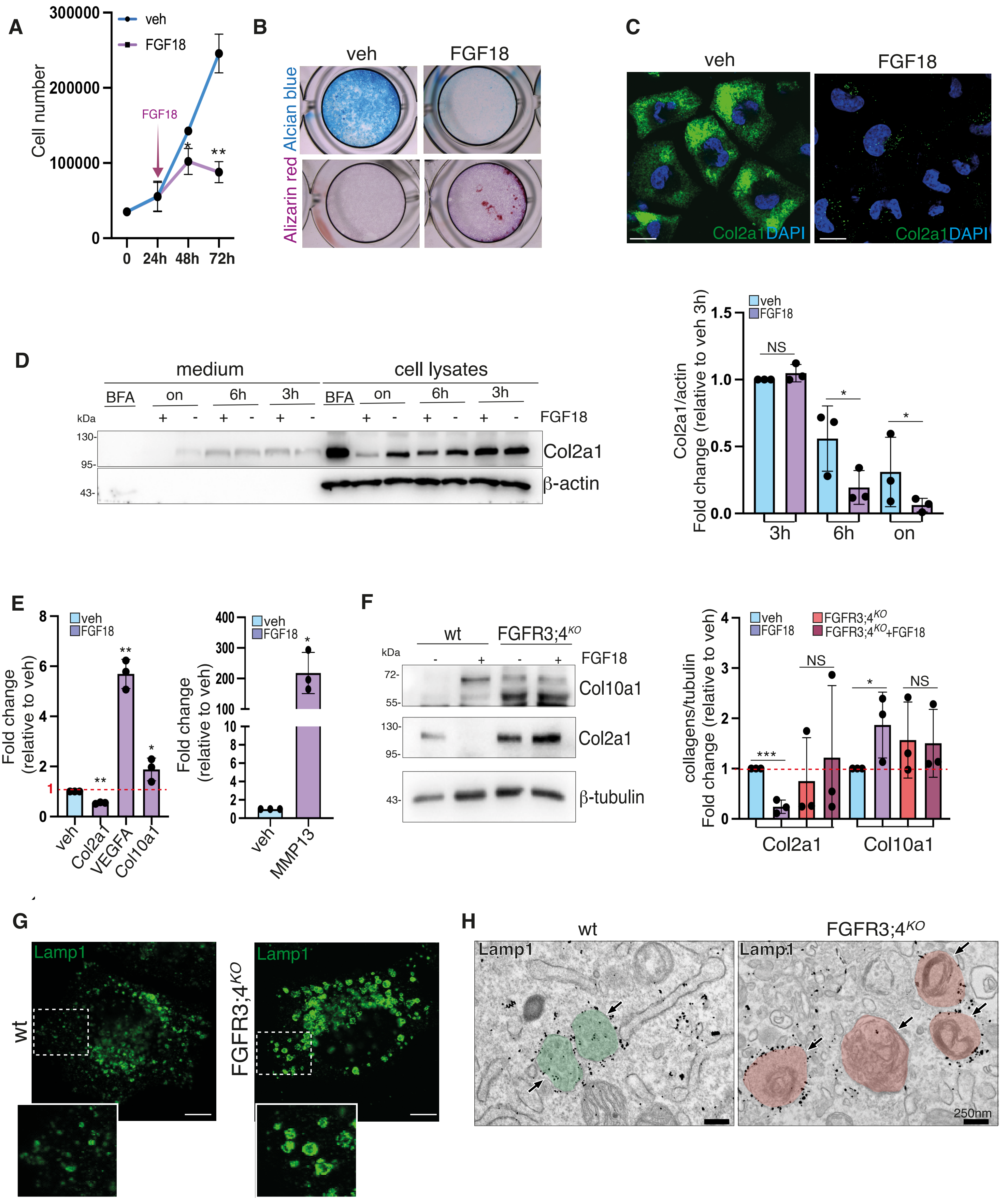

A
B
C
D
E
F
G
H
veh
FGF18
Cell number
Alcian blue
Alizarin red
Col2a1DAPI
medium
cell lysates
BFA
Col2a1
β-actin
Col2a1/actin
Fold change (relative to veh 3h)
NS
wt
FGFR3;4KO
Col10a1
β-tubulin
collagens/tubulin
Fold change (relative to veh)
VEGFA
MMP13
Lamp1
250nm

Figure 2

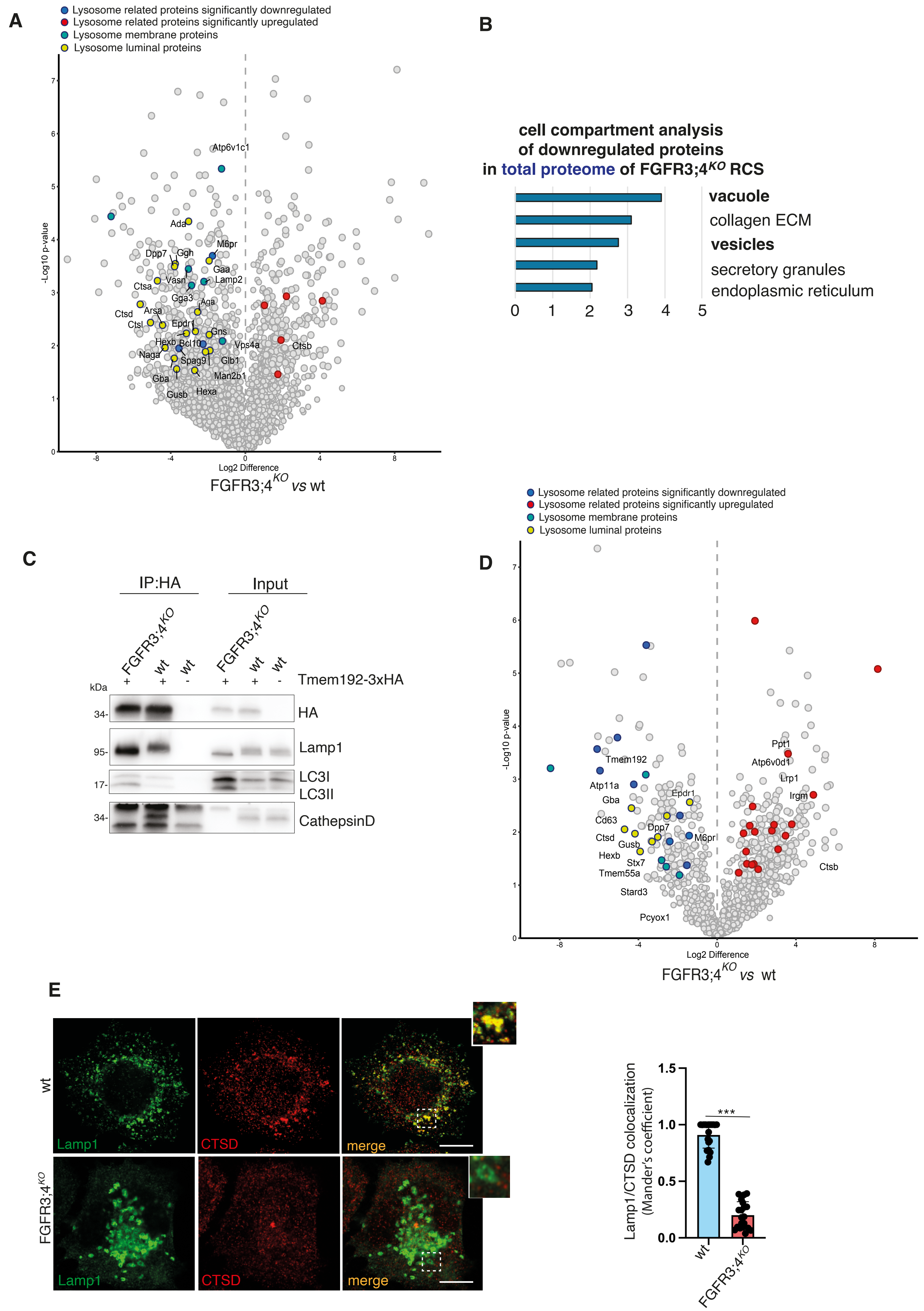

A
Lysosome related proteins significantly downregulated
Lysosome related proteins significantly upregulated
Lysosome membrane proteins
Lysosome luminal proteins
-Log10 p-value
Log2 Difference
FGFR3;4^{KO} vs wt
Atp6v1c1
Ada
M6pr
Dpp7
Ggh
Gaa
Vasn
Lamp2
Ctsa
Gga3
Aga
Ctsd
Arsa
Ctsl
Epdr1
Gns
Hexb
Bcl10
Vps4a
Ctsb
Naga
Spag9
Glb1
Gba
Man2b1
Gusb
Hexa
B
cell compartment analysis
of downregulated proteins
in total proteome of FGFR3;4^{KO} RCS
vacuole
collagen ECM
vesicles
secretory granules
endoplasmic reticulum
C
IP:HA
Input
FGFR3;4^{KO}
wt
Tmem192-3xHA
kDa
HA
Lamp1
LC3I
LC3II
CathepsinD
D
Lysosome related proteins significantly downregulated
Lysosome related proteins significantly upregulated
Lysosome membrane proteins
Lysosome luminal proteins
-Log10 p-value
Log2 Difference
FGFR3;4^{KO} vs wt
Ppt1
Tmem192
Atp6v0d1
Lrp1
Atp11a
Epdr1
Irgm
Gba
Cd63
Dpp7
M6pr
Ctsd
Gusb
Hexb
Stx7
Ctsb
Tmem55a
Stard3
Pcyox1
E
wt
FGFR3;4^{KO}
Lamp1
CTSD
merge
Lamp1/CTSD colocalization (Mander's coefficient)
***

Figure 3

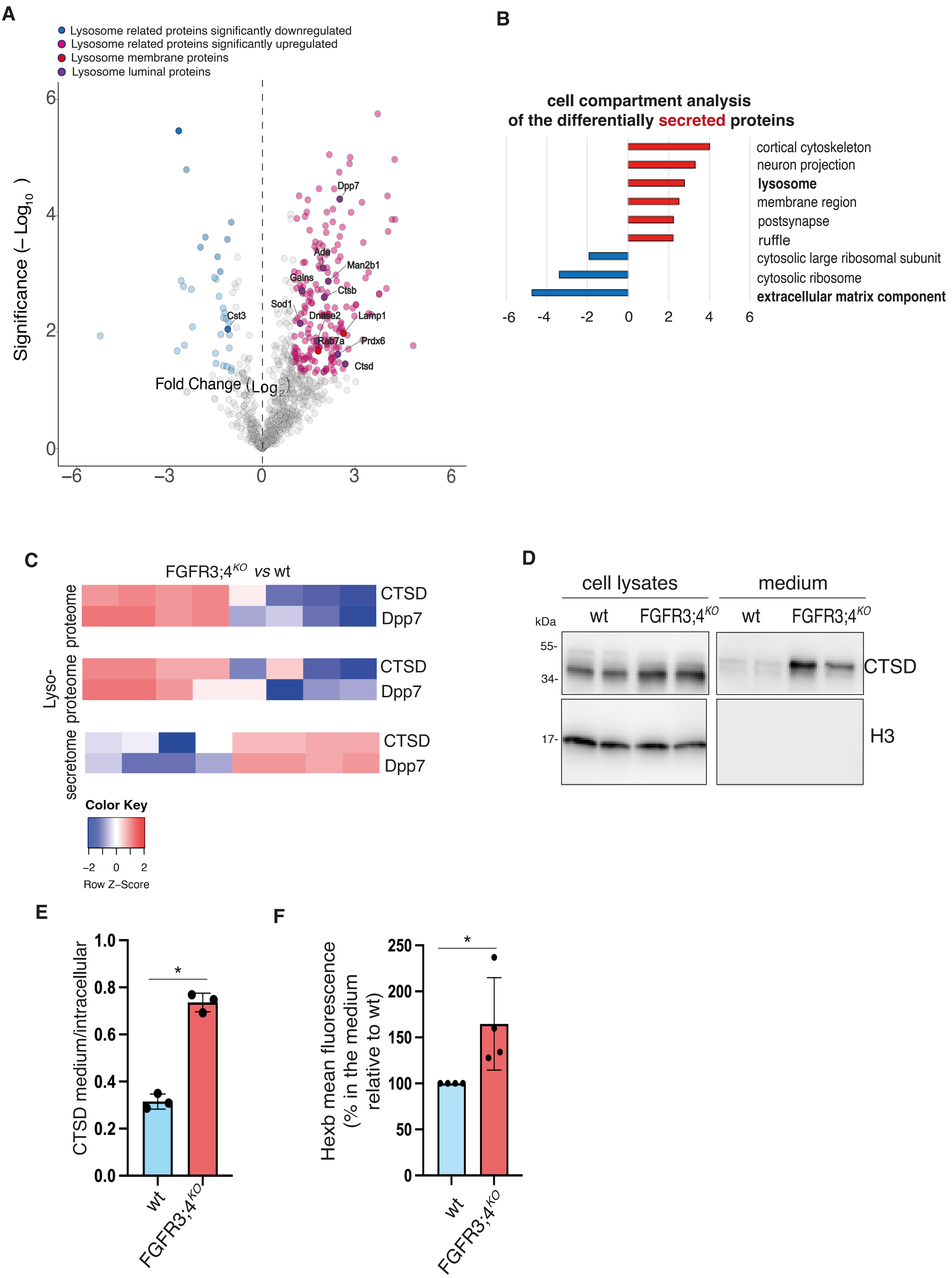
A
Lysosome related proteins significantly downregulated
Lysosome related proteins significantly upregulated
Lysosome membrane proteins
Lysosome luminal proteins
Significance (– Log10)
Fold Change (Log2)
Dpp7
Ada
Man2b1
Galns
Ctsb
Sod1
Cst3
Dnase2
Lamp1
Rab7a
Prdx6
Ctsd
B
cell compartment analysis
of the differentially secreted proteins
cortical cytoskeleton
neuron projection
lysosome
membrane region
postsynapse
ruffle
cytosolic large ribosomal subunit
cytosolic ribosome
extracellular matrix component
C
FGFR3;4KO vs wt
proteome
Lyso-proteome
secretome
CTSD
Dpp7
Color Key
Row Z–Score
D
cell lysates
medium
kDa
wt
FGFR3;4KO
55-
34-
17-
CTSD
H3
E
CTSD medium/intracellular
wt
FGFR3;4KO
F
Hexb mean fluorescence (% in the medium relative to wt)
wt
FGFR3;4KO

Figure 4

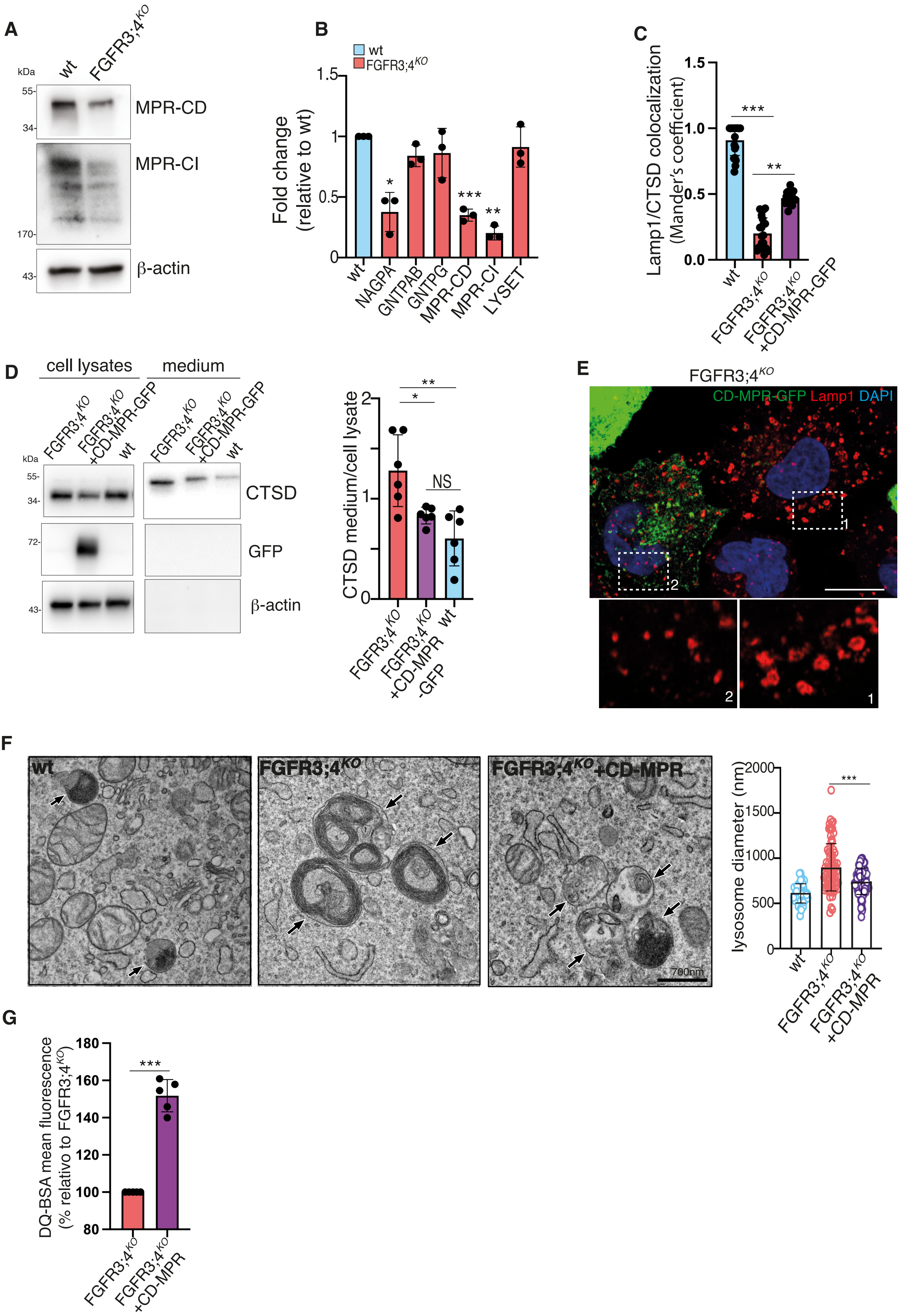
A
kDa
wt
FGFR3;4KO
55-
34-
MPR-CD
MPR-CI
170-
43-
β-actin
B
wt
FGFR3;4KO
Fold change
(relative to wt)
1.5
1
0.5
0
wt
NAGPA
GNTPAB
GNTPG
MPR-CD
MPR-CI
LYSET
C
Lamp1/CTSD colocalization
(Mander's coefficient)
1.5
1.0
0.5
0.0
wt
FGFR3;4KO
FGFR3;4KO
+CD-MPR-GFP
D
cell lysates
medium
FGFR3;4KO
FGFR3;4KO
+CD-MPR-GFP
wt
kDa
55-
34-
72-
43-
CTSD
GFP
β-actin
CTSD medium/cell lysate
2
1
0
NS
FGFR3;4KO
FGFR3;4KO
+CD-MPR
-GFP
E
FGFR3;4KO
CD-MPR-GFP Lamp1 DAPI
1
2
F
wt
FGFR3;4KO
FGFR3;4KO+CD-MPR
700nm
lysosome diameter (nm)
2000
1500
1000
500
0
G
DQ-BSA mean fluorescence
(% relativo to FGFR3;4KO)
180
160
140
120
100
80

Figure 5

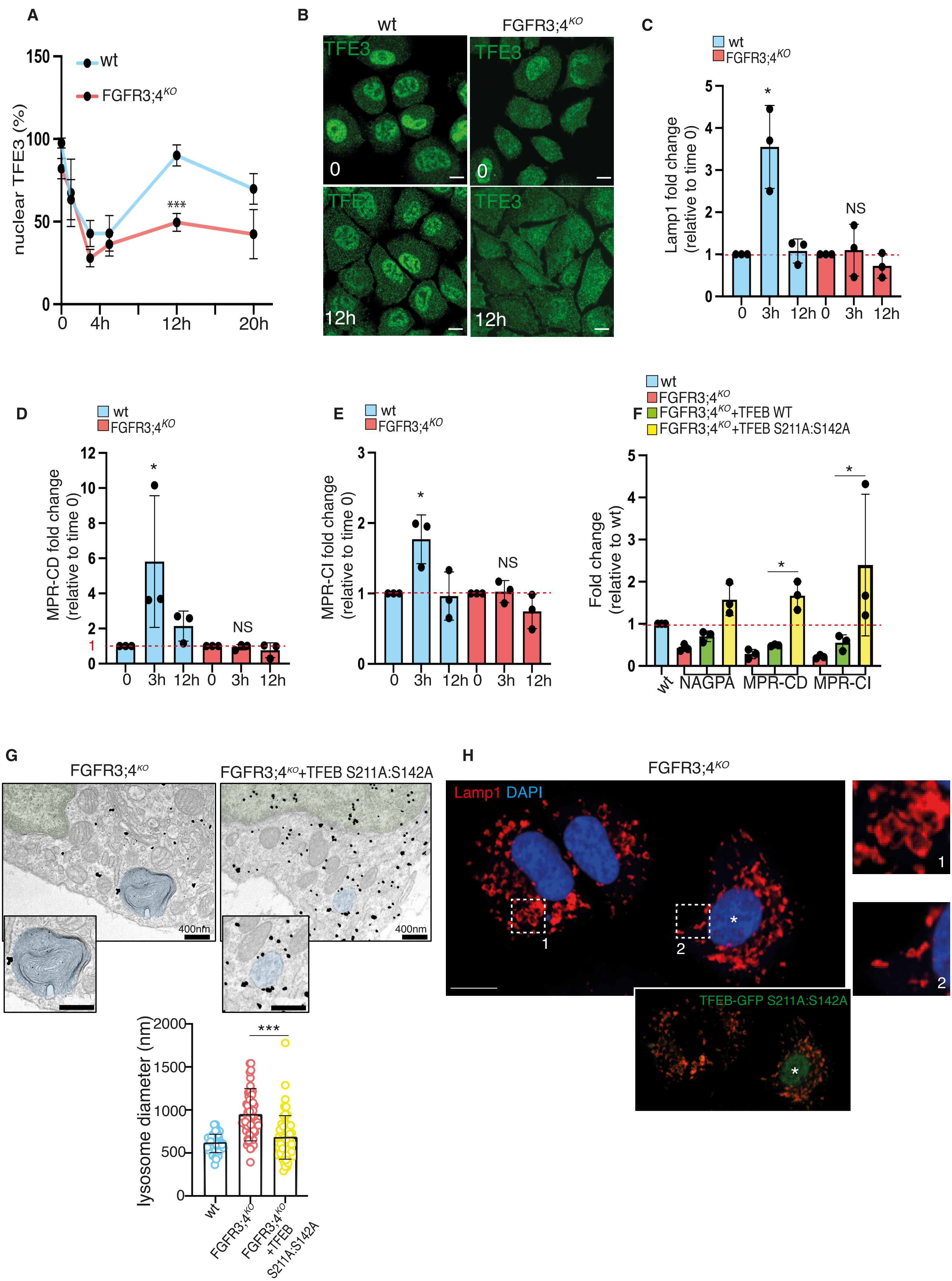

A
nuclear TFE3 (%)
wt
FGFR3;4KO
***
0
4h
12h
20h
B
wt
FGFR3;4KO
TFE3
0
12h
C
Lamp1 fold change (relative to time 0)
NS
D
MPR-CD fold change (relative to time 0)
E
MPR-CI fold change (relative to time 0)
F
FGFR3;4KO+TFEB WT
FGFR3;4KO+TFEB S211A:S142A
Fold change (relative to wt)
NAGPA
MPR-CD
MPR-CI
G
FGFR3;4KO
FGFR3;4KO+TFEB S211A:S142A
400nm
lysosome diameter (nm)
H
Lamp1 DAPI
TFEB-GFP S211A:S142A

Supplementary figure 1

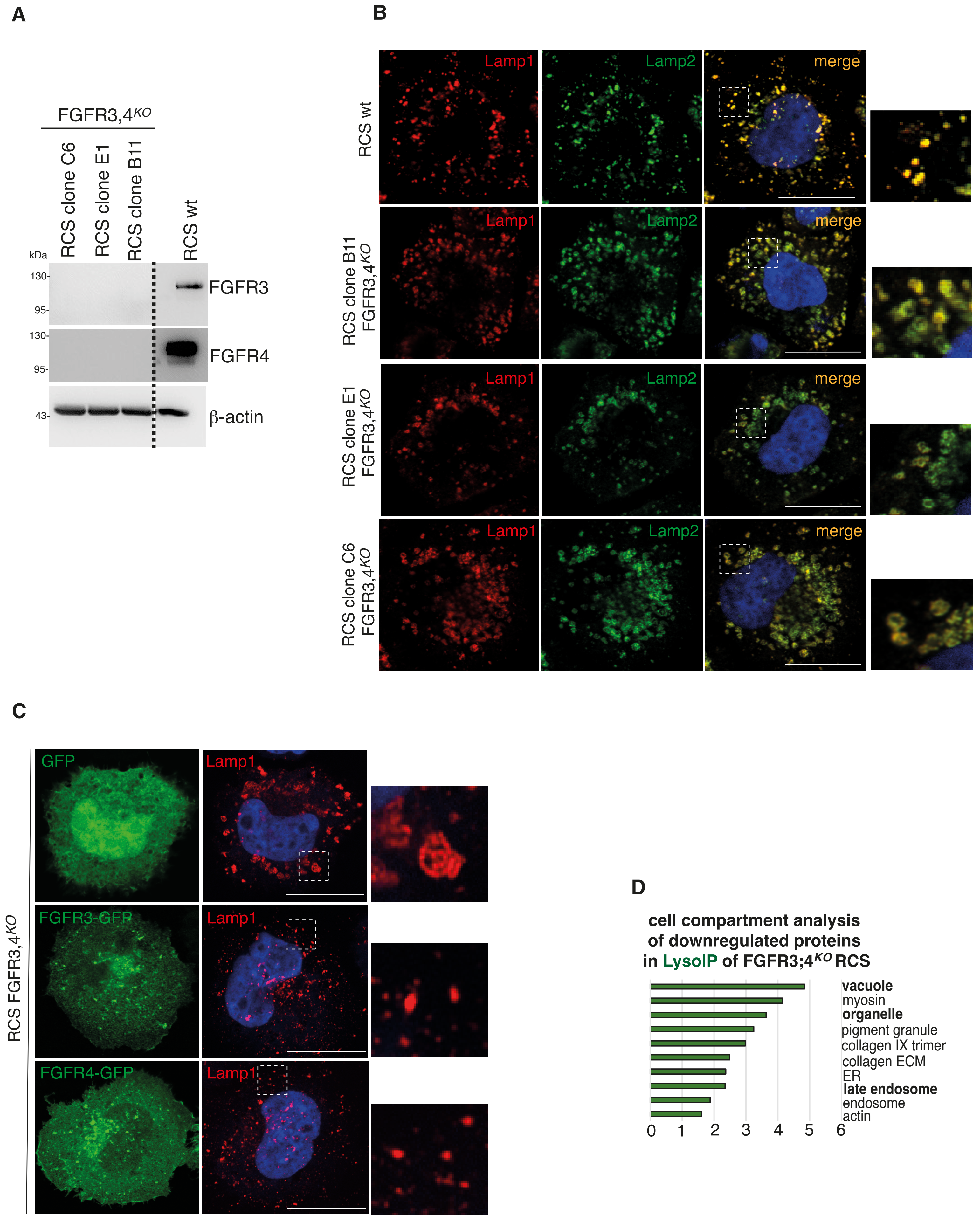

Supplementary figure 2

A

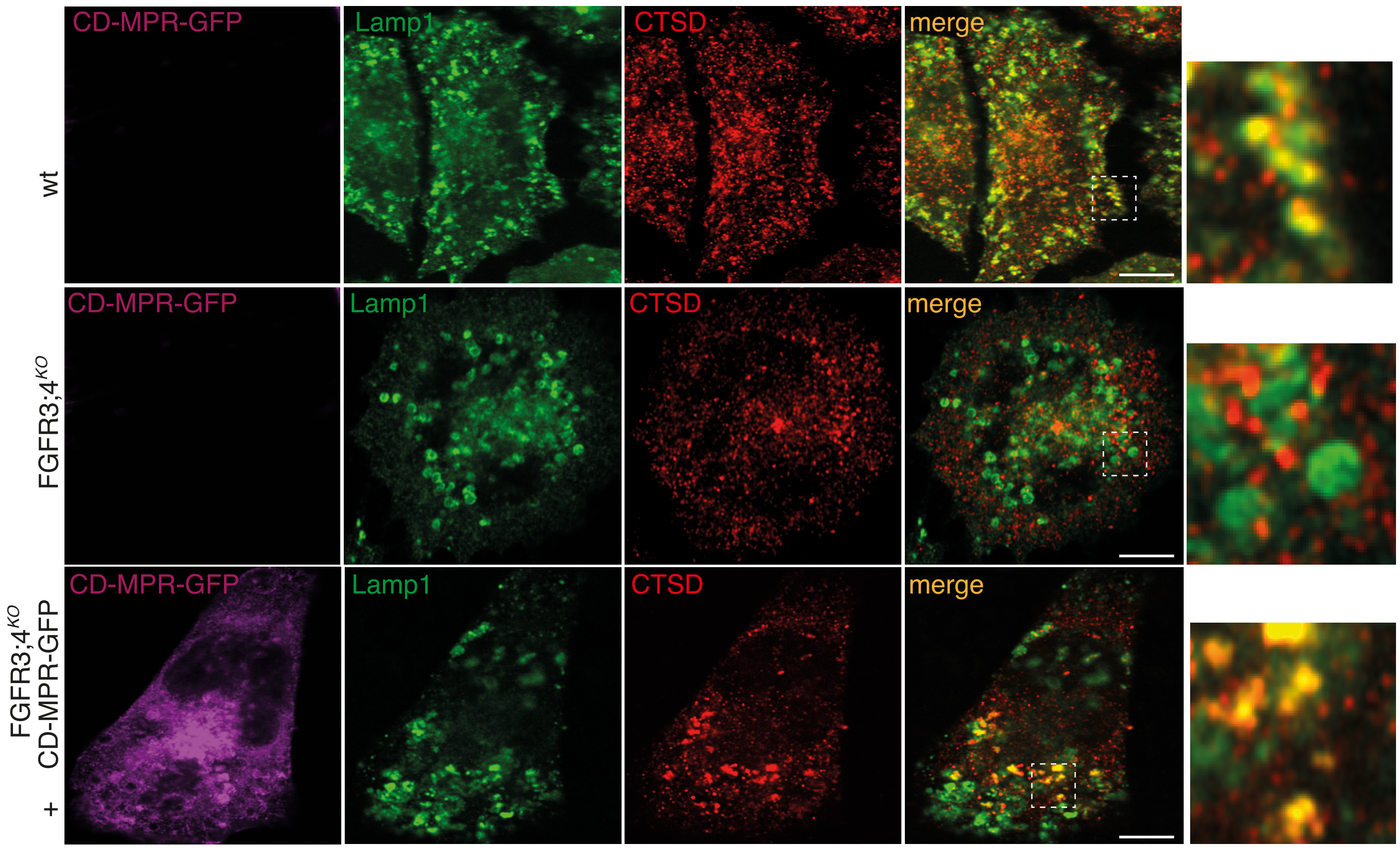

B

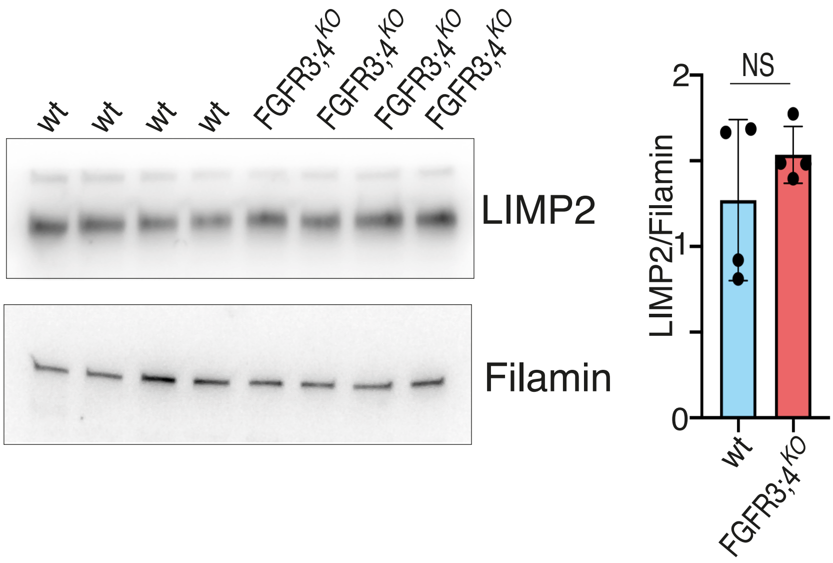

Supplementary figure 3

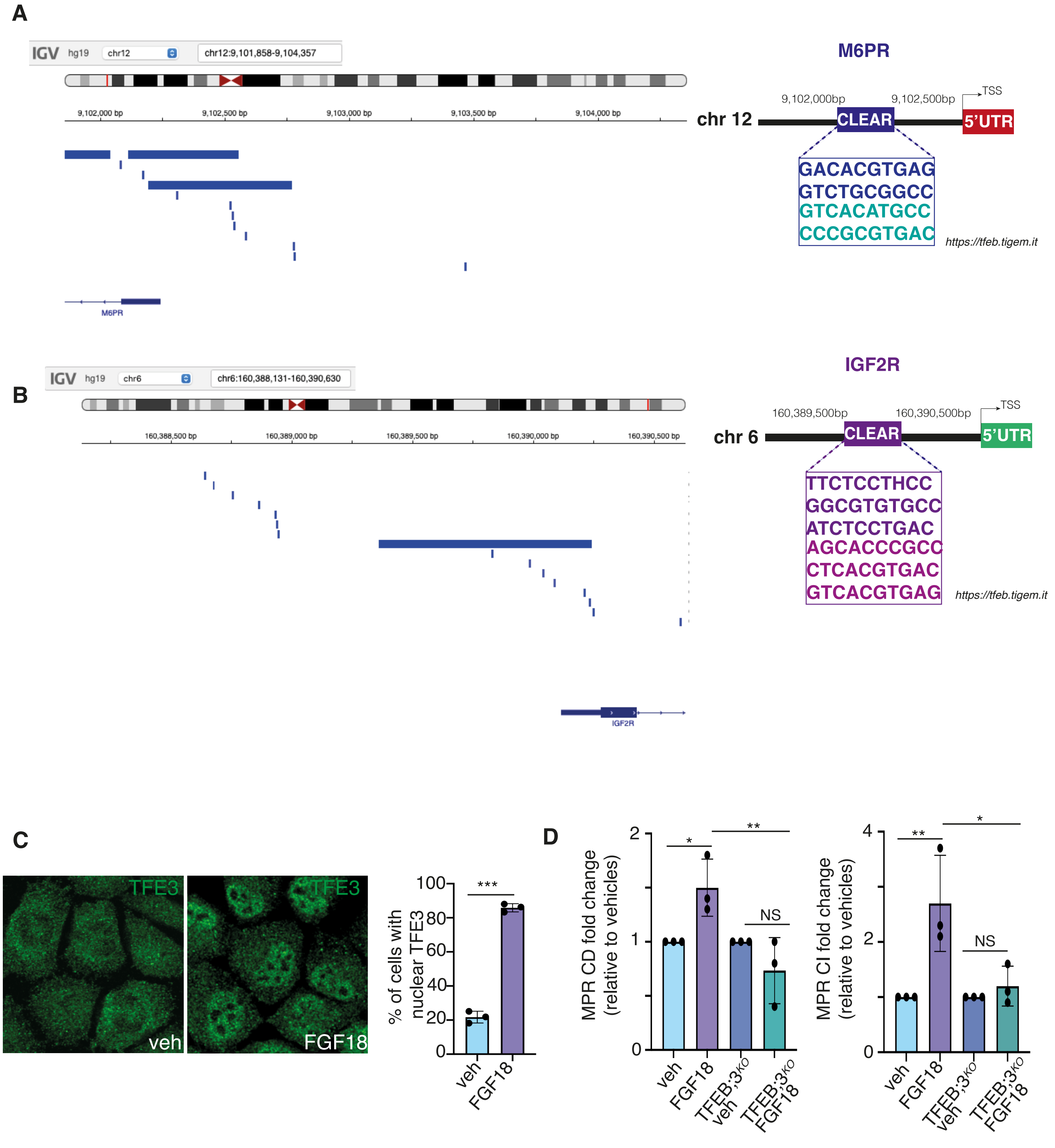

Supplementary figure 4

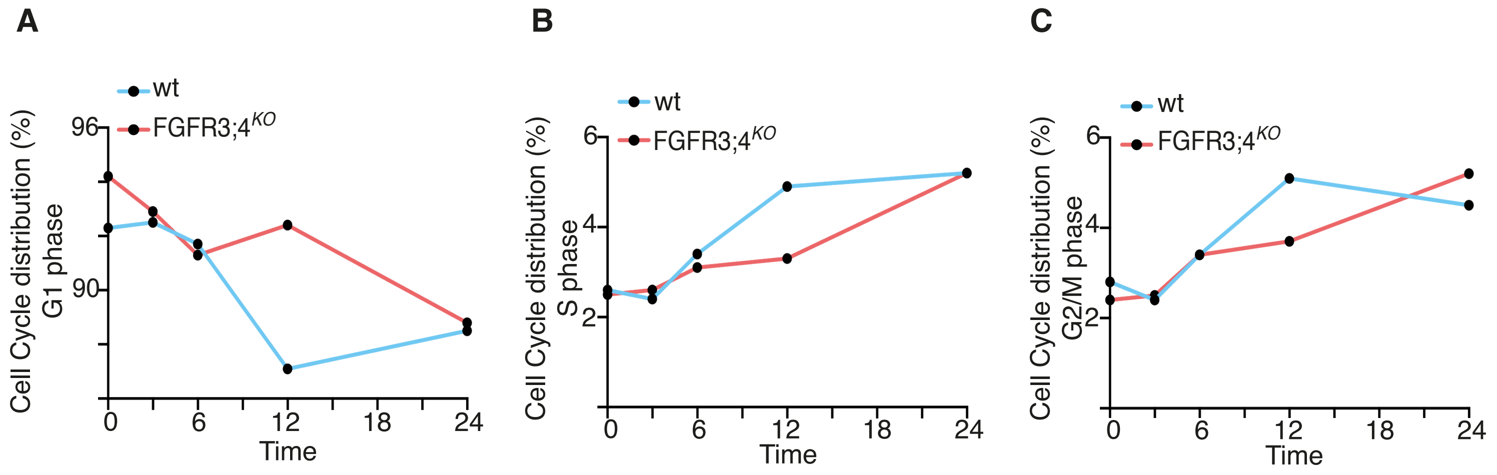

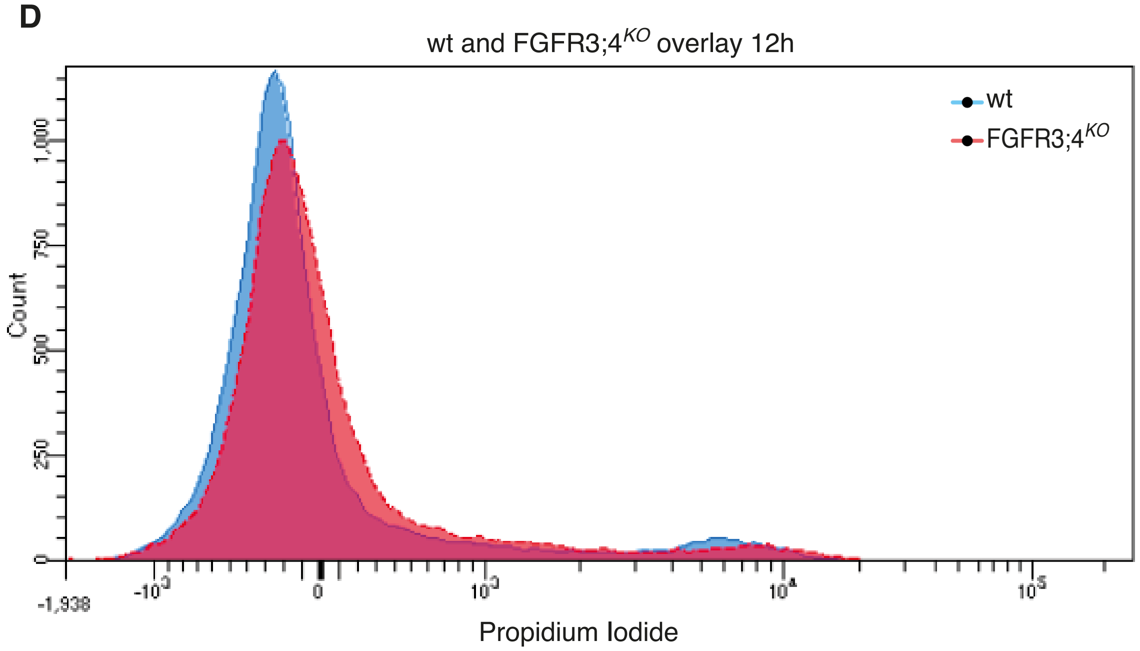

Supplement: Supplementary file 1 — Figure S1. (A) Western blot analysis of FGFR3 and FGFR4 protein levels in RCS wild type (wt) and FGFR3;4KO RCS clones. Beta‐actin was used as loading control. (B) Immunofluorescence analysis of Lamp1 (red) and Lamp2 (green) in RCS wild type (wt) and FGFR3;4KO RCS clones. DAPI (blue) was used to stain nuclei. Scale bar 10 μm. (C) Immunofluorescence analysis of Lamp1 (red) in RCS FGFR3;4KO transfected with pEGFP, FGFR3‐GFP and FGFR4‐GFP plasmids (green). Insets showed higher magnification of lysosomes. DAPI (blue) was used to stain nuclei. Scale bar 10 μm. (D) Cellular Compartment (CC) enrichment for the downregulated expressed proteins from Figure 2D. Figure S2. (A) Immunofluorescence staining of Lamp1 (green) and Cathepsin D (CTSD, red) in wt RCS, RCS FGFR3;4KO, and RCS FGFR3;4KO overexpressing CD‐MPR‐GFP (magenta). Magnification of the boxed areas showing lysosome and Cathepsin D colocalization are shown on the right. Scale bar 10 μm. (B) Western blot analysis of LIMP2 protein in RCS wt and RCS FGFR3;4KO showing no significant differences. N = 4 biological replicates. FilaminA was used as loading control. Student unpaired T‐test NS not significant. Figure S3. (A, B) IGV snapshots showing TFEB binding sites (indicated in dark blue) on the M6PR and IGF2R promoters, as identified by ChIP‐seq analysis [6]. Graphical representation of TFEB‐binding site sequence in the promoter of M6pr and Igf2r gene from Chip‐seq experiment [25]. (C) Immunofluorescence staining of TFE3 in RCS wt treated with vehicle (5% ABS) and FGF18 (50 ng/mL overnight). Bar graph represents the quantification of % of cells with nuclear TFE3. ± sem; Unpaired Student’s T‐test ***p < 0.0005. n = 3 biological replicates. (D) qPCR of Mpr‐cd and Mpr‐ci genes in RCS wild type (wt) and TFEB;3KO RCS treated with vehicle (5% ABS) or FGF18 (50 ng/mL, overnight). N = 3 biological replicates ± sem. One‐way ANOVA, Sidàk’s multiple comparison test *p < 0.05; **p < 0.005; NS not significant. Figure S4. (A‐C) Quantit [file TRA-26-e70013-s007.pdf]
